# Risks and benefits of oral anticoagulants for stroke prophylaxis in atrial fibrillation according to body mass index: Nationwide cohort study of primary care records in England

**DOI:** 10.1016/j.eclinm.2022.101709

**Published:** 2022-10-31

**Authors:** Yoko M. Nakao, Kazuhiro Nakao, Jianhua Wu, Ramesh Nadarajah, A. John Camm, Chris P. Gale

**Affiliations:** a Leeds Institute of Cardiovascular and Metabolic Medicine, University of Leeds, Leeds, UK; b Leeds Institute for Data Analytics, University of Leeds, Leeds, UK; c School of Dentistry, University of Leeds, Leeds, UK; d Department of Cardiology, Leeds Teaching Hospitals NHS Trust, Leeds, UK; e Molecular and Clinical Sciences Research Institute, St George's University of London, London, UK

**Keywords:** Atrial fibrillation, Direct oral anticoagulants, Body mass index, Stroke, Bleeding

## Abstract

**Background:**

Direct oral anticoagulants (DOACs) are effective and safe alternatives to warfarin for stroke prophylaxis for atrial fibrillation (AF). Whether this extends to patients at the extremes of body mass index (BMI) is unclear.

**Methods:**

Using linked primary and secondary data, Jan 1, 2010 to Nov 30, 2018, we included CHA_2_DS_2_-VAS_C_ score ≥3 in women and ≥2 in men with AF treated with oral anticoagulants (OACs). Outcomes were ischaemic stroke, major bleeding and all-cause mortality by World Health Organisation BMI classification. Patients who received warfarin were propensity score matched (1:1 ratio) with those who received DOACs and the association of time-varying OAC exposure on outcomes quantified using Cox proportional hazards models.

**Findings:**

We included 29,135 (22,818 warfarin, 6317 DOAC); 585 (2.0%) underweight, 8427 (28.9%) normal weight, 10,705 (36.7%) overweight, 5910 (20.3%) class I obesity and 3508 (12.0%) class II/III obesity. Patients treated with DOACs were older and more comorbid. After 3.7 (SD 2.5) years follow up, there was no difference in risk of ischaemic stroke and major bleeding by BMI category between DOACs and warfarin. Normal weight, overweight and obese class I patients had higher risk of all-cause mortality when treated with DOACs compared with warfarin (HR: 1.45 [95% CI 1.24–1.69], *p* < 0.001; 1.41 [95% CI 1.19–1.66], *p* < 0.001; and 1.90 [95% CI 1.50–2.39], *p* < 0.001), an effect not observed after DOACs became the most common OAC prescription. Amongst underweight patients OAC exposure was associated with greater harm from bleeding than benefit from stroke prevention (benefit to harm ratio, 0.35 [95% CI 0.26–0.44]).

**Interpretation:**

In patients with AF in each BMI classification we found no difference in ischaemic stroke and bleeding risk for DOACs compared with warfarin. Underweight patients experienced divergent risk-benefit patterns from oral anticoagulation compared with other BMI categories.

**Funding:**

None.

**Research in context**

**Evidence before this study**

We searched Medline and Embase for reports published in English from inception to March 2022 with a combination of keywords and subject headings related to atrial fibrillation (AF), body mass index (BMI) and oral anticoagulants (warfarin, direct oral anticoagulants [DOACs]). We also reviewed reference lists of selected reports.

Randomised controlled trials (RCTs) demonstrated that DOACs were safer and effective alternatives to warfarin for stroke prophylaxis for patients with AF. However, patients with very low or very high BMI constituted a small proportion of those recruited. The effect of DOACs is dependent on plasma concentration so the risks and benefits of DOACs may alter at the extremes of BMI. Observational studies have either not been generalizable, only examined one of the extremes of BMI, or had few patients at the extremes. Many authors have performed intention-to-treat analyses or switch-censored, and so have not captured the full extent of oral anticoagulant exposure. We found no European population-based study that provided a robust analysis of DOACs compared with warfarin across the range of BMI.

**Added value of this study**

Our study provides novel information on the risks and benefits of DOACs and warfarin for stroke prophylaxis for AF according to BMI strata in a large general population cohort in England. We demonstrate that between 2010 and 2018 warfarin prescription declined whilst DOAC prescription increased. Moreover, patients treated with DOACs were older and more comorbid than those treated with warfarin. We provide reassuring evidence that in routine clinical practice there is no difference in the risk of bleeding and risks of stroke between treatment with DOACs and treatment with warfarin in each BMI category, including at the extremes of BMI. We also provide evidence that patients who fail to persist with oral anticoagulation are at increased risk of ischaemic stroke and all-cause mortality. Finally, comparing the benefit of stroke prevention to harm from bleeding, we demonstrate that underweight patients experience a worse outcome profile to patients in other BMI categories.

**Implications of all the available evidence**

The number of people who are extremely obese or underweight is growing and this population is commonly at increased risk of AF and stroke. Novel RCTs to bolster the evidence base at the extremes of BMI are unlikely given that DOACs are nearing patent end. Across European, Korean and American populations observational data in real-world populations suggests that DOACs are safe and effective alternatives to warfarin in AF patients with very high or very low BMI for stroke prophylaxis. Underweight patients with AF are subject to higher rates of adverse outcomes and the risk-benefit profile for oral anticoagulation in this group may not be clear-cut, thereby requiring tailored individual-level clinical decision making.

## Introduction

Direct oral anticoagulants (DOACs) are safe and effective alternatives to warfarin for stroke prophylaxis for patients with atrial fibrillation (AF),^1^ and have superseded warfarin for this indication in national and international guidelines.^2^^,^^3^ Patients at the extremes of body weight (BMI [body mass index] <18.5 kg/m^2^ and BMI ≥35 kg/m^2^) were under-represented in trials,^4^ and constitute an increasing proportion of the population.^5^ BMI is an important variable in drug distribution and plasma concentrations, but DOACs are mostly prescribed without therapeutic drug monitoring—this raises concerns that the safety and efficacy of DOACs may be compromised at the extremes of BMI.^4^

Thus far, observational studies have provided an incomplete picture (Supplementary Table S1); some only investigated people with obesity,^6^^,^^7^ others only investigated underweight populations.^8^ One study reported a comparison across BMI strata but only from one hospital system.^9^ Moreover, studies have either censored at switching,^6^ or conducted an intention-to-treat analysis,^8^^,^^10^ thereby not taking into account the full exposure of a patient to oral anticoagulants (OACs) if they switch agent or investigating how failing to persist with OAC affects the risk of later events; even though long-term persistence is low in real-world practice.^11^

To address this knowledge gap, we assessed the risks (major bleeding and mortality) and benefits (reduced ischaemic stroke) associated with DOACs compared with warfarin in patients with AF at elevated stroke risk across the range of BMI, including the extremes of weight, using a nationwide cohort of linked primary and secondary care records.

## Methods

### Study design and setting

We conducted this population-based, retrospective cohort study using the Clinical Practice Research Datalink-GOLD (CPRD-GOLD). CPRD-GOLD contains anonymised patient data from about 7% of the UK population and is largely representative of the UK population in terms of age, sex and ethnicity.^12^ Primary care records from CPRD were linked to secondary care admission records from Hospital Episode Statistics Admitted Patient Care data (HES-APC) and cause-specific mortality from the Office for the National Statistics (ONS).

### Ethics statement

This study based in part on data from the CPRD which has ethics approval from the Health Research Authority to support research using anonymised patient data. Scientific approval for this study was given by the CPRD Independent Scientific Advisory Committee (ref no: 19_076).

### Study population

The study period was 1st January 2010 to 30th November 2018. We included patients aged 18 years or older with a new diagnosis of AF, defined as at least one clinical or referral event in CPRD-GOLD or International Classification of Diseases version 10 [ICD-10] code in HES-APC, and at least one prescription of OAC. We excluded patients without OAC prescription after AF diagnosis and patients in receipt of a prescription for an OAC in the 120 days before the first OAC prescription after AF diagnosis. We also excluded those with an error for their first OAC prescription date, those without follow-up after diagnosis of AF and those without any measurements of height and weight or implausible measurements (weight <30 kg or >300 kg, height <1 m or >2.5 m). BMI was calculated directly from the most recent weight and height record relative to the date that the patients were first prescribed an OAC (weight/height^2^). Cases where BMI was recorded at the same time or soon after the first OAC prescription were included as OAC prescription itself does not promote weight management behaviour and thus it is unlikely to have a significant influence on BMI measurement. The end of follow-up was defined as the earliest of occurrence of an outcome, death, transfer out of a contributing practice, the last collection date of a contributing practice, or the study end date.

### Exposure to anticoagulants

Exposure to OACs was defined as the receipt of prescription for an OAC after receiving a diagnosis of AF. The drug index date was the date of the first prescription of an OAC after the diagnosis of AF in the study period. Gaps in prescribing of <90 days were considered as on treatment because this a timeframe that reflects the typical maximum duration of an OAC prescription issued in UK primary care (Supplementary Figure S1). Exposure was modelled as a time-varying variable, allowing patients to switch between different OAC exposure groups during the follow-up period or to not persist with OACs. We defined non-persistence as the period when there was a gap in prescribing of an OAC for ≥90 days. This allowed an interpretation of the risk of not receiving OAC prescription in individuals at elevated risk of stroke. OACs included warfarin and the four DOACs—dabigatran, rivaroxaban, apixaban, and edoxaban. In the presence of overlap of two different medications (that is, a switch in therapy from warfarin to a DOAC or from a DOAC to warfarin), the overlapped portion was credited to the latter medication. Patients co-prescribed OACs and antiplatelets were included the study cohort, and we adjusted for the use of antiplatelets in the multivariate analyses. The daily dose was categorised as standard (300 mg for dabigatran, 20 mg for rivaroxaban, 10 mg for apixaban, and 60 mg for edoxaban) or lower than the recommended daily dose.

### Outcomes

The outcomes were ischaemic stroke, major bleeding and all-cause mortality after diagnosis of AF. Ischaemic stroke was based on CPRD, HES, and ONS codes. We included unclassified strokes within ischaemic strokes because ∼87% of all strokes are ischaemic.^13^ Major bleeding included intracranial haemorrhage and gastrointestinal bleeding which led to a hospital admission or death, based on HES and ONS codes. The date of outcomes was the earliest record after entry into the study from primary care, hospital and mortality data records after index date of OAC prescription.

### Covariates

Covariates included demographic and lifestyle variables (age at index date, sex, smoking status), deprivation (index of multiple deprivation [IMD] quintiles), comorbidities (heart failure, hypertension, diabetes mellitus, myocardial infarction, peripheral artery disease, stroke, transient ischaemic attack, chronic obstructive pulmonary disease [COPD], chronic kidney disease [CKD], gastrointestinal bleeding, cancer, dementia and depression), and current medications prescribed at the index date (angiotensin-converting enzyme inhibitor/angiotensin-receptor blocker, beta-blockers, amiodarone, statins, proton-pump inhibitors, corticosteroids, non-steroidal anti-inflammatory drugs and antiplatelets). Each covariate was chosen either because it is used as an indicator for prescribing a specific OAC or because it is associated with increased risk of ischaemic stroke or bleeding.

### Statistical analysis

A primary analysis was conducted in patients with a CHA_2_DS_2_-VAS_C_ score ≥3 in women and ≥2 in men, because guidelines give a class I recommendation for OACs for such patients.^3^ We categorised BMI (underweight: <18.5 kg/m^2^, normal range: 18.5–24.9 kg/m^2^, overweight: 25.0–29.9 kg/m^2^, obese class I 30.0–34.9 kg/m^2^, obese class II/III: ≥35.0 kg/m^2^) according to the World Health Organisation (WHO) classification.^14^ Patients with missing ethnicity data were included in the white category.^15^ Patients with missing smoking data were included in the non-smoker category. We used propensity score matching with the covariates listed above to adjust for potential confounding from imbalances in clinical characteristics between patients treated with warfarin and DOACs in each BMI group. Propensity scores were estimated using logistic regression after excluding missing IMD data (n = 6) (Supplementary Table S4). Patients who received warfarin were matched in a 1:1 ratio with those who received DOACs using nearest neighbour matching without replacement with a calliper of 0.2 standard deviation (SD) (Supplementary Methods). Differences in clinical characteristics were assessed using standardised differences. Baseline characteristics for patients, by BMI categories and OAC, were described as percentages or mean (SD) as appropriate.

We calculated incidence rates expressed as per 1000 person years of follow-up for outcomes. We used Kaplan–Meier curves to visualise the cumulative incidence in patients with and without OACs by BMI categories. We assessed the association of time-varying OAC exposure on the outcomes using Cox proportional hazards models stratified by BMI with adjustment for covariates. For ischaemic stroke and major bleeding informative censoring of survival time was taken into account for those who died as a competing risk using Fine and Gray's proportional sub-hazards model,^16^ to estimate cause-specific hazard ratios (HRs) and 95% confidence intervals (CIs). We developed a predictive model to determine the benefit to harm ratio of OACs versus time without OACs (‘Off OACs’) considering that OACs might be accompanied by additional, clinically significant, serious adverse events, using a method similar to that of Phillips et al. (Supplementary Methods).^17^

We assessed the sensitivity of analytical method and inclusion criteria. To provide a more direct comparison to the methodology of previous studies we conducted analyses by intention-to-treat and switch-censoring.^8^ We also investigated whether results would be altered by 1) only using weight measured prior to the first OAC prescription (up to 3 years),^18^ 2) restricting the window period between prescriptions to 60 days, 3) excluding patients with a preceding interventional procedure for AF or stroke prophylaxis (e.g. ablation, left atrial appendage closure, surgical left atrial appendage removal), and 4) conducting multiple imputation of missing data. Finally, given that in 2010 warfarin was the most common OAC and from 2015 prescription rates for DOACs were higher,^15^ with patient characteristics differing by OAC type and over time, we ran a sensitivity analysis including only patients with a OAC index date from 1st January 2015 onwards.

We performed an analysis including patients with CHA_2_DS_2_-VAS_C_ score ≥2 in women and ≥1 in men, to understand if our findings extended to this group who are eligible for OACs but at lower stroke risk.^3^ We also performed an analysis stratified by standard dose and lower dose in the DOAC group.

Analyses were performed using Stata version 16 (Stata Corp., College Station, TX, USA). All statistical tests were two-sided with a *p* value <0.05 considered to be significant. Study findings are reported in accordance with the Reporting of studies Conducted using Observational Routinely-collected health Data (RECORD) recommendations.^19^

### Role of the funding source

None. All authors had full access to all the data in the study and accept responsibility to submit for publication.

## Results

A total of 17,578,233 patients contributed data from 1st January 2010 to 30th November 2018. After application of inclusion and exclusion criteria 29,135 patients formed the analytical cohort before matching (Supplementary Figure S2). Of those patients, 22,818 were prescribed warfarin and 6317 were prescribed DOACs (dabigatran 579 [2.0%], rivaroxaban 2970 [9.8%], apixaban 2617 [9.0%], edoxaban 151 [0.5%]). The overall mean (SD) age was 77.6 (8.5) years, 13,148 patients (45.1%) were women and mean CHA_2_DS_2_-VASc score was 3.6 (1.3) in men and 4.6 (1.3) in women. According to the WHO classification of BMI, 585 (2.0%) patients were underweight, 8427 (28.9%) were normal weight, 10,705 (36.7%) were overweight, 5910 (20.3%) had class I obesity and 3508 (12.0%) had class II/III obesity.

**Fig. 1:**
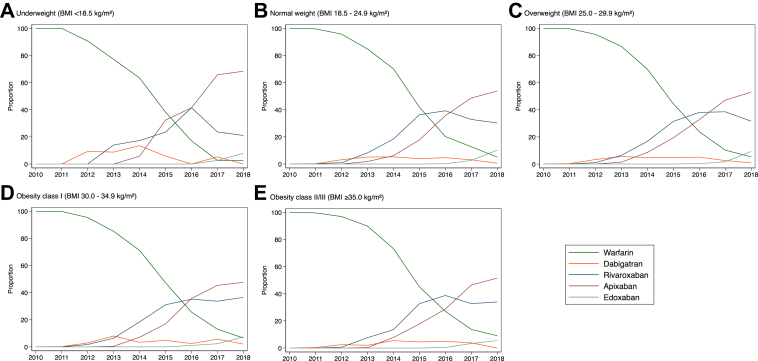
Proportion of patients prescribed different OAC first in each year by BMI. OACs = oral anticoagulants; BMI = body mass index.

Fig. 1 shows that overall 78.3% of patients were prescribed warfarin, but initial prescription of warfarin declined during the study period from 95.7% in 2012 to 5.9% in 2018 with a concurrent increase in prescription of DOACs. By the end of the study period, apixaban was the most frequently prescribed OAC across all BMI categories. Amongst those prescribed warfarin, 420 (1.8%) were underweight and 2724 (11.9%) had obesity class II/III; whilst amongst patients prescribed DOACs 165 (2.6%) were underweight and 784 (12.4%) had class II/III obesity. Patients prescribed DOACs were older (78.4 years vs. 77.5, *p* < 0.001) and more commonly women (46.4% vs. 44.8%, *p* = 0.024) compared with those treated with warfarin. They also less frequently had heart failure (19.7% vs. 26.2%, *p* < 0.001) and CKD (31.9% vs. 34.7%, *p* < 0.001) and more frequently had hypertension (83.7% vs. 81.1%, *p* < 0.001), diabetes mellitus (29.8% vs. 26.4%, *p* < 0.001), stroke (19.7% vs. 17.5%, *p* < 0.001), gastrointestinal bleeding (15.7% vs. 14.4%, *p* = 0.013), cancer (26.9% vs. 23.3%, *p* < 0.001), dementia (6.7% vs. 2.0%, *p* < 0.001) and depression (26.9% vs. 22.6%, *p* < 0.001) (Supplementary Table S2).

**Fig. 2:**
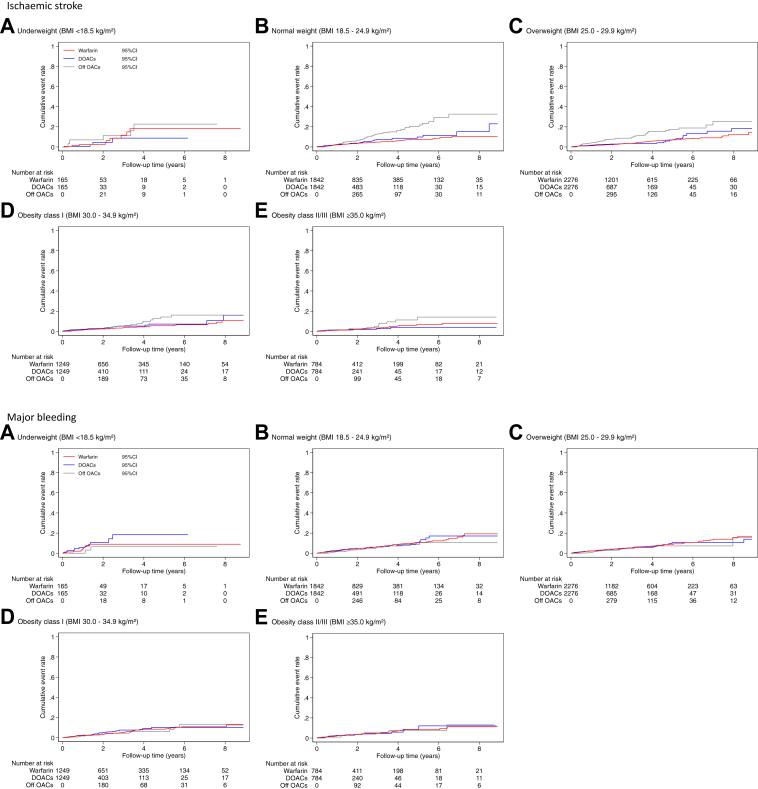

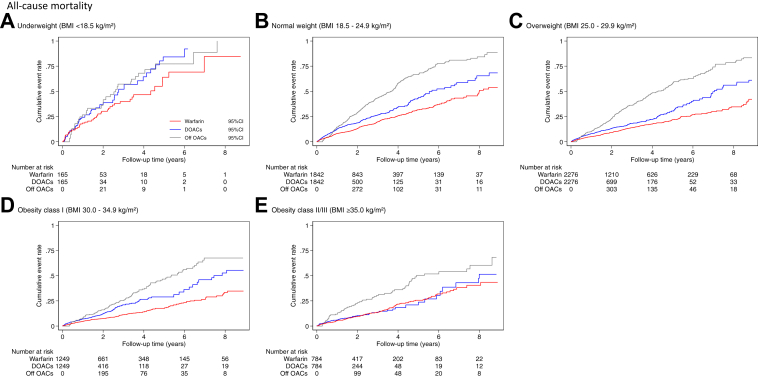
Kaplan–Meier survival curves for outcomes by BMI and OAC type. “Off OACs” refers to patients who were prescribed OACs but did not persist with prescriptions. OACs = oral anticoagulants; BMI = body mass index; DOACs = direct oral anticoagulants; CI = confidence interval.

Before matching, patients with a higher BMI category tended to be younger, more commonly men, more frequently had heart failure, hypertension, diabetes mellitus, CKD and depression and less frequently had previous stroke, COPD, gastrointestinal bleeding, cancer and dementia (Supplementary Table S3). In the propensity score matched cohort of 6316 pairs, we found no major differences between the two groups (Table 1, Supplementary Table S5). The mean (SD) duration of follow up was 3.7 (2.5) years. The crude incidence rates of ischaemic stroke, major bleeding and all-cause mortality are presented in Table 2. In general, underweight patients had higher incidence rates for each outcome compared to other BMI categories. For the full study period, the incidence rates for ischaemic stroke and major bleeding were similar between patients treated with DOACs or warfarin across BMI categories, and for all-cause mortality were generally higher in patients taking DOACs. For patients who failed to persist with OACs (Off OACs), the incidence rates of ischaemic stroke and all-cause mortality were higher than those who persisted and this was consistent across all BMI categories. The cumulative event rate curves for each outcome are shown in Fig. 2.

**Table 1:** Baseline characteristics in patients with anticoagulants stratified by BMI and OAC type at the study entry after propensity score matching.

BMI categories	Warfarin					DOACs				
	Underweight (<18.5 kg/m^2^)	Normal weight (18.5–24.9 kg/m^2^)	Overweight (25.0–29.9 kg/m^2^)	Obese class I (30.0–34.9 kg/m^2^)	Obese class II/III (≥35.0 kg/m^2^)	Underweight (<18.5 kg/m^2^)	Normal weight (18.5–24.9 kg/m^2^)	Overweight (25.0–29.9 kg/m^2^)	Obese class I (30.0–34.9 kg/m^2^)	Obese class II/III (≥35.0 kg/m^2^)
No of patients	165	1842	2276	1249	784	165	1842	2276	1249	784
Mean age (SD)	83.6 (6.7)	81.6 (6.8)	78.2 (7.6)	76.3 (8.0)	72.8 (8.9)	83.5 (8.0)	81.6 (8.2)	78.5 (8.9)	76.1 (9.0)	72.8 (9.1)
Women	115 (69.7%)	947 (51.4%)	957 (42.0%)	521 (41.7%)	391 (49.9%)	112 (67.9%)	936 (50.8%)	945 (41.5%)	531 (42.5%)	406 (51.8%)
Ethnicity (White)	163 (98.8%)	1818 (98.7%)	2201 (96.7%)	1220 (97.7%)	768 (98.0%)	163 (98.8%)	1815 (98.5%)	2209 (97.1%)	1226 (98.2%)	771 (98.3%)
IMD										
1 (Affluent)	44 (26.7%)	577 (31.3%)	698 (30.7%)	322 (25.8%)	165 (21.0%)	49 (29.7%)	564 (30.6%)	689 (30.3%)	321 (25.7%)	155 (19.8%)
2	35 (21.2%)	383 (20.8%)	519 (22.8%)	260 (20.8%)	136 (17.3%)	32 (19.4%)	404 (21.9%)	506 (22.2%)	237 (19.0%)	152 (19.4%)
3	24 (14.5%)	420 (22.8%)	448 (19.7%)	282 (22.6%)	151 (19.3%)	29 (17.6%)	405 (22.0%)	463 (20.3%)	289 (23.1%)	156 (19.9%)
4	31 (18.8%)	260 (14.1%)	343 (15.1%)	197 (15.8%)	196 (25.0%)	29 (17.6%)	271 (14.7%)	350 (15.4%)	203 (16.3%)	177 (22.6%)
5 (Deprived)	31 (18.8%)	202 (11.0%)	268 (11.8%)	188 (15.1%)	136 (17.3%)	26 (15.8%)	198 (10.7%)	268 (11.8%)	199 (15.9%)	144 (18.4%)
Current or ex-smoker	98 (59.4%)	1048 (56.9%)	1345 (59.1%)	799 (64.0%)	489 (62.4%)	99 (60.0%)	1054 (57.2%)	1374 (60.4%)	798 (63.9%)	488 (62.2%)
Heart failure	36 (21.8%)	344 (18.7%)	404 (17.8%)	243 (19.5%)	205 (26.1%)	37 (22.4%)	341 (18.5%)	431 (18.9%)	245 (19.6%)	192 (24.5%)
Hypertension	119 (72.1%)	1431 (77.7%)	1912 (84.0%)	1105 (88.5%)	719 (91.7%)	121 (73.3%)	1450 (78.7%)	1890 (83.0%)	1105 (88.5%)	722 (92.1%)
DM	21 (12.7%)	347 (18.8%)	583 (25.6%)	470 (37.6%)	414 (52.8%)	21 (12.7%)	367 (19.9%)	610 (26.8%)	469 (37.6%)	416 (53.1%)
MI	19 (11.5%)	248 (13.5%)	275 (12.1%)	191 (15.3%)	129 (16.5%)	20 (12.1%)	263 (14.3%)	323 (14.2%)	202 (16.2%)	115 (14.7%)
PAD	19 (11.5%)	144 (7.8%)	158 (6.9%)	89 (7.1%)	60 (7.7%)	20 (12.1%)	153 (8.3%)	173 (7.6%)	101 (8.1%)	61 (7.8%)
Stroke	24 (14.5%)	381 (20.7%)	490 (21.5%)	250 (20.0%)	123 (15.7%)	24 (14.5%)	391 (21.2%)	468 (20.6%)	243 (19.5%)	119 (15.2%)
TIA	12 (7.3%)	170 (9.2%)	230 (10.1%)	135 (10.8%)	58 (7.4%)	11 (6.7%)	178 (9.7%)	230 (10.1%)	137 (11.0%)	58 (7.4%)
COPD	57 (34.5%)	395 (21.4%)	401 (17.6%)	263 (21.1%)	190 (24.2%)	59 (35.8%)	410 (22.3%)	422 (18.5%)	266 (21.3%)	180 (23.0%)
CKD	51 (30.9%)	513 (27.9%)	709 (31.2%)	413 (33.1%)	258 (32.9%)	52 (31.5%)	542 (29.4%)	728 (32.0%)	438 (35.1%)	252 (32.1%)
GI bleeding	30 (18.2%)	282 (15.3%)	344 (15.1%)	203 (16.3%)	116 (14.8%)	29 (17.6%)	302 (16.4%)	343 (15.1%)	203 (16.3%)	113 (14.4%)
Cancer	60 (36.4%)	548 (29.8%)	631 (27.7%)	306 (24.5%)	160 (20.4%)	61 (37.0%)	556 (30.2%)	626 (27.5%)	301 (24.1%)	153 (19.5%)
Dementia	18 (10.9%)	136 (7.4%)	125 (5.5%)	51 (4.1%)	20 (2.6%)	23 (13.9%)	159 (8.6%)	156 (6.9%)	61 (4.9%)	25 (3.2%)
Depression	49 (29.7%)	445 (24.2%)	522 (22.9%)	357 (28.6%)	274 (34.9%)	49 (29.7%)	458 (24.9%)	546 (24.0%)	360 (28.8%)	287 (36.6%)
ACEI/ARB	60 (36.4%)	813 (44.1%)	1218 (53.5%)	732 (58.6%)	520 (66.3%)	59 (35.8%)	819 (44.5%)	1228 (54.0%)	743 (59.5%)	503 (64.2%)
Beta-blockers	15 (9.1%)	176 (9.6%)	252 (11.1%)	141 (11.3%)	98 (12.5%)	12 (7.3%)	167 (9.1%)	245 (10.8%)	143 (11.4%)	90 (11.5%)
Amiodarone	7 (4.2%)	49 (2.7%)	63 (2.8%)	44 (3.5%)	13 (1.7%)	7 (4.2%)	55 (3.0%)	75 (3.3%)	48 (3.8%)	19 (2.4%)
Statins	56 (33.9%)	869 (47.2%)	1245 (54.7%)	743 (59.5%)	490 (62.5%)	57 (34.5%)	888 (48.2%)	1291 (56.7%)	754 (60.4%)	484 (61.7%)
PPIs	64 (38.8%)	752 (40.8%)	889 (39.1%)	514 (41.2%)	344 (43.9%)	65 (39.4%)	781 (42.4%)	935 (41.1%)	521 (41.7%)	359 (45.8%)
Corticosteroids	28 (17.0%)	180 (9.8%)	198 (8.7%)	126 (10.1%)	118 (15.1%)	24 (14.5%)	177 (9.6%)	182 (8.0%)	111 (8.9%)	103 (13.1%)
NSAIDs	1 (0.6%)	30 (1.6%)	63 (2.8%)	27 (2.2%)	26 (3.3%)	1 (0.6%)	37 (2.0%)	70 (3.1%)	32 (2.6%)	28 (3.6%)
Anti-platelets	60 (36.4%)	881 (47.8%)	1074 (47.2%)	648 (51.9%)	38 (49.1%)	57 (34.5%)	863 (46.9%)	1089 (47.8%)	660 (52.8%)	367 (46.8%)

BMI = body mass index; SD = standard deviation; IMD = index of multiple deprivation; DOACs = direct oral anticoagulants; DM = diabetes mellitus; MI = myocardial infarction; OAC = oral anticoagulant; PAD = peripheral artery disease; TIA = transient ischaemic attack; COPD = chronic obstructive pulmonary disease; CKD = chronic kidney disease; GI = gastrointestinal; ACEI/ARB = angiotensin-converting enzyme inhibitor/angiotensin receptor blocker; PPIs = proton pump inhibitors; NSAIDs = non-steroidal anti-inflammatory drugs.

Figures are n (%) unless otherwise stated. Current medication use was prescribed within the last 90 days. Standardized differences before and after propensity-matching shows in the Supplementary Table S5.

**Table 2:** Incidence rates per 1000 person years and 95% confidence intervals of outcomes by body mass index categories.

	Whole^a^	Underweight (<18.5 kg/m^2^)	Normal weight (18.5–24.9 kg/m^2^)	Overweight (25.0–29.9 kg/m^2^)	Obese class I (30.0–34.9 kg/m^2^)	Obese class II/III (≥35.0 kg/m^2^)
Ischaemic stroke						
Warfarin	14.4 (12.6–16.3)	32.2 (16.8–61.9)	16.4 (13.0–20.7)	14.2 (11.5–17.5)	12.1 (8.9–16.3)	11.9 (8.1–17.7)
DOACs	17.5 (15.1–20.3)	20.3 (17.8–29.4)	22.9 (17.8–29.4)	15.5 (11.9–20.1)	18.3 (13.3–25.1)	10.3 (6.0–17.8)
Off OACs	37.1 (31.3–43.9)	52.2 (21.7–125.4)	48.8 (37.3–63.9)	38.6 (29.1–51.2)	24.1 (15.2–38.2)	22.8 (12.3–42.3)
Major bleeding						
Warfarin	20.6 (18.5–22.9)	33.3 (17.4–64.1)	24.3 (20.0–29.4)	19.8 (16.6–23.7)	18.3 (14.3–23.5)	17.3 (12.5–23.9)
DOACs	22.3 (19.6–25.5)	61.5 (34.9–108.2)	23.9 (18.7–30.6)	20.2 (16.1–25.4)	22.7 (17.0–30.2)	18.3 (12.1–27.5)
Off OACs	19.1 (15.0–24.4)	23.5 (5.9–93.8)	20.1 (13.0–31.1)	18.3 (11.9–28.0)	18.5 (10.7–31.8)	19.3 (9.7–38.6)
All-cause mortality						
Warfarin	59.2 (55.6–63.1)	182.3 (138.5–239.8)	76.8 (69.0–85.5)	50.9 (45.6–56.9)	43.0 (36.7–50.4)	58.3 (48.9–69.6)
DOACs	88.6 (83.0–94.6)	268.3 (205.5–350.3)	116.1 (104.0–129.7)	76.7 (68.2–86.2)	75.0 (64.2–87.6)	58.6 (46.7–73.5)
Off OACs	177.2 (164.1–191.3)	308.3 (215.6–440.9)	224.5 (198.3–254.1)	173.5 (152.1–198.0)	126.5 (103.7–154.3)	128.6 (99.4–166.4)

DOACs = direct oral anticoagulants; OACs = oral anticoagulants.

“Off OACs” refers to patients who were prescribed oral anticoagulants (OACs) but did not persist with prescriptions. The number of events is shown in Supplementary Table S6.

a Whole refers to total patients after propensity-score matching by each body mass index categories (n = 12632).

Compared with warfarin there was no significant difference in risk of ischaemic stroke or bleeding by BMI category for patients prescribed DOACs (Table 3). For patients taking DOACs, the risk of all-cause mortality was not different to warfarin in underweight or obese class II/III patients, but was higher in patients who were normal weight, overweight, and obese class I (HR: 1.45 [95% CI 1.24–1.69] *p* < 0.001; 1.41 [95% CI 1.19–1.66], *p* < 0.001; and 1.90 [95% CI 1.50–2.39], *p* < 0.001).

**Table 3:** Hazard ratios and 95% confidence intervals by BMI categories.

BMI categories	Underweight (<18.5 kg/m^2^)		Normal weight (18.5–24.9 kg/m^2^)		Overweight (25.0–29.9 kg/m^2^)		Obese class I (30.0–34.9 kg/m^2^)		Obese class II/III (≥35.0 kg/m^2^)	
	HR (95% CI)	*p*	HR (95% CI)	*p*	HR (95% CI)	*p*	HR (95% CI)	*p*	HR (95% CI)	*p*
Ischaemic stroke										
DOACs	0.49 (0.09–2.80)	0.422	1.10 (0.77–1.56)	0.614	0.93 (0.66–1.32)	0.688	1.26 (0.79–2.02)	0.331	0.68 (0.33–1.41)	0.299
Off OACs	1.52 (0.34–6.87)	0.585	2.51 (1.72–3.66)	<0.001	2.41 (1.62–3.57)	<0.001	2.05 (1.16–3.62)	0.014	1.94 (0.83–4.51)	0.125
Major bleeding										
DOACs	1.48 (0.55–3.96)	0.432	0.84 (0.61–1.16)	0.298	0.86 (0.64–1.15)	0.310	1.04 (0.71–1.53)	0.853	0.99 (0.58–1.71)	0.982
Off OACs	0.70 (0.13–3.78)	0.676	0.70 (0.43–1.16)	0.165	0.76 (0.48–1.22)	0.265	0.88 (0.48–1.61)	0.671	1.19 (0.51–2.77)	0.686
All-cause mortality										
DOACs	1.37 (0.91–2.05)	0.134	1.45 (1.24–1.69)	<0.001	1.41 (1.19–1.66)	<0.001	1.90 (1.50–2.39)	<0.001	0.99 (0.74–1.34)	0.972
Off OACs	1.80 (1.09–2.99)	0.023	2.47 (2.08–2.92)	<0.001	3.16 (2.65–3.77)	<0.001	2.76 (2.12–3.58)	<0.001	2.16 (1.56–3.00)	<0.001

BMI = body mass index; HR = hazard ratio; CI = confidence interval; DOACs = direct oral anticoagulants; OACs = oral anticoagulants.

The reference is warfarin. These were adjusted for age, gender, ethnicity, index of multiple deprivation, smoking, heart failure, hypertension, diabetes mellitus, myocardial infarction, peripheral artery disease, stroke, transient ischaemic attack, chronic kidney disease, gastrointestinal bleeding, cancer, dementia, depression, angiotensin converting enzyme inhibitor/angiotensin receptor blocker, beta-blockers, amiodarone, statins, proton-pump inhibitors, corticosteroids, non-steroidal anti-inflammatory drugs, and anti-platelets. “Off OACs” refers to patients who were prescribed oral anticoagulants (OACs) but did not persist with prescriptions.

Periods of non-persistence with OACs were associated with higher risks of ischaemic stroke (HR: normal weight 2.51 [95% CI 1.72–3.66], *p* < 0.001; overweight 2.41 [95% CI 1.62–3.57], *p* < 0.001; obese class I 2.05 [95% CI 1.16–3.62], *p* = 0.014), except at the extremes of BMI where wide CIs led to statistical non-significance (HR: underweight: 1.52 [95% CI 0.34–6.87], *p* = NS; obese class II/III: 1.94 [95% CI 0.83–4.51], *p* = NS). They were also associated with a higher risk of all-cause mortality (HR: underweight 1.80 [95% CI 1.09–2.99], *p* = 0.023; normal weight 2.47 [95% CI 2.08–2.92], *p* < 0.001; overweight 3.16 [95% CI 2.65–3.77], *p* < 0.001; obese class I 2.76 [95% CI 2.12–3.58], *p* < 0.001; obese class II/III 2.16 [95% CI 1.56–3.00], *p* < 0.001). In benefit to harm analysis we found that exposure to OACs, as opposed to time without OAC prescription, was associated with benefit (ratio >1.0, indicating positive net benefit) across individuals who were normal weight (1.71 [95% CI 1.64–1.78], overweight (2.01 [95% CI 1.91–2.11]) and obese (1.92 [95% CI 1.65–2.20]; Supplementary Figure S3). By contrast, an inverse relationship was found in underweight patients (0.35 [95% CI 0.26–0.44]).

The results were not altered in the intention-to-treat and switch-censored analyses, when weight measured up to 3 years prior to first OAC prescription was used, when multiple imputation was conducted for missing data, when patients with a preceding interventional AF-related procedure were excluded, and when the prescription period was shortened to 60 days (Supplementary Table S10). The analyses that incorporated AF patients with lower stroke risk (CHA_2_DS_2_-VAS_C_ score ≥2 in women and ≥1 in men) and stratified standard and lower dose of DOACs also agreed with the main analysis. However, when restricted to patients with an OAC index date from 1st January 2015 onwards, when DOACs became the most common anticoagulant prescription, there was no difference in the risk of all-cause mortality between DOACs and warfarin across normal weight, overweight, obese class I, and obese class II/III patients, with insufficient data for analysis of underweight patients.

## Discussion

In this nationwide study of patients with AF at elevated risk of stroke, we found that the risk of major bleeding and ischaemic stroke were similar for warfarin and DOAC treatment across WHO BMI classifications. Although we found the risk of all-cause mortality was higher for patients prescribed DOACs in the early part of the study period, there was no difference once the use of DOACs became common place. Exposure to oral anticoagulation, compared to periods without OAC prescription, was associated with a lower risk ischaemic stroke and all-cause mortality, but in underweight patients the benefit of stroke risk reduction was outweighed by an increased bleeding risk.

Novel RCT evidence to address the use of DOACs compared with warfarin for stroke prophylaxis in AF patients with very low or very high BMI is unlikely given that many of the DOACs are nearing patent end.^20^ Previous population-based studies have either included only a very small number of underweight patients,^21^ only provided data for one DOAC,^22^ or included venous thromboembolism as an indication for prescription.^21^

Observational studies conducted in the United States of America have found a similar or lower risk for stroke/systemic embolism and major bleeding for apixaban, rivaroxaban and dabigatran compared with warfarin in obese cohorts.^6^^,^^7^ A report from a single American hospital system found risk reductions for ischaemic stroke (∼25%) and significant bleeding (∼50%) for DOAC recipients compared with patients prescribed warfarin across the BMI range.^9^ The authors reported incidence rates for ischaemic stroke and bleeding amongst patients treated with warfarin that were two to four times higher than in our population across each BMI category, whereas the incidence rates amongst patients treated with DOAC were very similar to our own. Notably, their analytical cohort was not propensity-score matched and patients treated with warfarin were older and more comorbid.

A Korean study of underweight patients (body weight <60 kg) found better effectiveness and safety for DOACs over warfarin at both regular and reduced on-label dosing, whereas we found no difference.^8^ However, that study was in an ethnically uniform Asian cohort who, compared with people of European ancestry, are generally of lower weight and increased risk of stroke and bleeding.^4^ In our analysis underweight patients, compared with patients in other BMI strata, were at higher risk of adverse outcomes, and in these patients oral anticoagulation exposure was associated with a greater increase in risk of major bleeding compared with reduction in risk of ischaemic stroke. Low body weight is commonly associated with frailty, malnutrition, cancer, heart failure, CKD and increased risk of falling; all of which can synergistically increase the risk of bleeding.^23^ Although our observation is at risk of residual confounding from unmeasured factors, this analysis highlights the complexities of clinical decision making in this high risk group, and the importance of managing predisposing pathologies (e.g. cancer) and modifiable risk factors (e.g. hypertension).^24^

We found that use of DOACS was associated with an increased risk of all-cause mortality in some BMI categories in the study period up to 2015, when DOAC use was less common. A UK study also found that the use of rivaroxaban and lower doses of apixaban between 2011 and 2016 was associated with an increased risk of all-cause mortality compared to warfarin.^15^ In our study patients prescribed DOACs rather than warfarin before 2015 were significantly older and had a higher prevalence of preceding stroke, dementia and concomitant antiplatelet use (Supplementary Table S9). Death in AF patients is most commonly as a result of diseases other than ischaemic stroke and bleeding,^25^ and a greater proportion of the older and more comorbid patients taking DOACs may have died from these causes while taking anticoagulation.

The strengths of this study include its sample size, a nationally representative population and the long duration of follow-up. The primary care records were linked to hospital and mortality data, so major outcomes were identified. We purposefully included patients with valvular heart disease, even though they were excluded from some observational studies, because a meta-analysis has shown that DOAC risks compared with warfarin were similar for patients with AF with and without valvular heart disease.^26^ Through implementation of propensity-score matching we ensured the DOAC and warfarin groups were well-balanced for covariates known to impact the risk of ischaemic stroke or bleeding. We accounted for mortality as a competing risk in the calculation of hazard ratios for stroke and bleeding and we modelled OAC exposure as a time-varying variable to more closely represent the risks and benefits of OACs in clinical practice. We confirmed the fidelity of our findings across different analytical methods.

Study limitations include its observational nature, meaning only statistical associations may be inferred, and that outcomes are based on clinical codes without further arbitration, which may lead to under- or over-estimation of incidence. Warfarin and DOACs had different time periods and reported associations may be confounded by drug indication. Our population was predominantly Caucasian and the ethnic composition of the cohort should be considered when these results are interpreted and generalised. Though we used prescription as a proxy of persistence with treatment, actual drug adherence could not be ascertained and we did not have information on why patients failed to persist with OAC prescription. Finally, we did not investigate the quality of warfarin treatment by time in therapeutic ratio (TTR), which has previously been estimated in routine UK clinical practice to be about 70%, with 25% of patients having a TTR of <65%.^27^ Nonetheless, the inclusion of patients with worse quality management of warfarin and off-label DOAC dosing enables a better understanding of the true risk and benefits of these medications in real-world practice.

In summary, this national primary care records study provides reassuring evidence that stroke and bleeding risk did not vary between DOACs and warfarin in patients with AF across all BMI classifications in routine clinical practice. Underweight patients were at elevated risk of adverse outcomes and were subject to divergent patterns of benefit and risk from oral anticoagulation compared to other BMI categories.

## Contributors

YMN, KN, JW, and CPG conceived and designed the research question. YMN, KN, and JW prepared the data for analysis. YMN and KN analysed the data. All authors (YMN, KN, JW, RN, AJC, and CPG) had access to and verified the underlying study data and responsibility for the decision to submit for publication. YMN and RN wrote the first draft of the manuscript. All authors (YMN, KN, JW, RN, AJC, and CPG) provided input on interpretation of results. All authors (YMN, KN, JW, RN, AJC, and CPG) revised the manuscript critically for important intellectual content and read and approved the final manuscript.

## Data sharing statement

CPRD data governance does not allow us to distribute or make available patient data directly to other parties. The diagnostic code lists used in this study are available on reasonable request of the corresponding author.

## Declaration of interests

YMN reports a study grant from Bayer. CPG reports personal fees from AstraZeneca, Amgen, Bayer, Boehrinher-Ingelheim, Daiichi Sankyo, Vifor, Pharma, Menarini, Wondr Medical, Raisio Group and Oxford University Press. He has received educational and research grants from BMS, Abbott Inc., the British Heart Foundation, National Institute of Health Research, Horizon 2020, and from the European Society of Cardiology, outside the submitted work. AJC reports personal fees from Abbott, Bayer, Daiichi Sankyo, Pfizer, BMS, Sanofi, Medtronic, Boston Scientific and Menarini. All other authors declare no competing interests.
