# Supplementary material for: Risks and benefits of oral anticoagulants for stroke prophylaxis in atrial fibrillation according to body mass index: Nationwide cohort study of primary care records in England
Source: eClinicalMedicine. 2022 Oct 31;54:101709. doi: 10.1016/j.eclinm.2022.101709 (PMC9637568; doi:10.1016/j.eclinm.2022.101709)
Supplement: Supplementary Material [file mmc1.docx]

Supplementary appendix

Risks and benefits of oral anticoagulants for stroke prophylaxis in atrial fibrillation according to body mass index: Nationwide cohort study of primary care records in England

Yoko M Nakao, MD · Kazuhiro Nakao, MD · Jianhua Wu, PhD · Ramesh Nadarajah, MA · A John Camm, MD · Chris P Gale, PhD

Table of Contents

[Part 1 – Additional Methods 2](#_Toc115264569)

[Exposure 2](#_Toc115264570)

[Covariates 2](#_Toc115264571)

[Propensity score matching and covariates 2](#_Toc115264572)

[Missing data and multiple imputation 2](#_Toc115264573)

[Benefit and harm analysis 3](#_Toc115264574)

[Part 2 – Additional Results 4](#_Toc115264575)

[Supplementary Table 1. Previous observational studies reporting risk and benefit of direct oral anticoagulants in atrial fibrillation patients across body mass index categories 4](#_Toc115264576)

[Supplementary Table 2. Baseline characteristics in whole cohort by oral anticoagulant type 6](#_Toc115264577)

[Supplementary Table 3. Baseline characteristics before matching, by BMI and oral anticoagulant type 7](#_Toc115264578)

[Supplementary Table 4. Coefficients in the propensity matching model 9](#_Toc115264579)

[Supplementary Table 5. Standardized differences before and after propensity-matching 10](#_Toc115264580)

[Supplementary Table 6. Number of events by body mass index categories 12](#_Toc115264581)

[Supplementary Table 7. Baseline characteristics in patients with CHA_2_DS_2_-VAS_C_ score ≥ 2 in women and ≥ 1 in men stratified by body mass index and oral anticoagulant type at the study entry after propensity score matching 13](#_Toc115264582)

[Supplementary Table 8. Baseline characteristics on lower and standard doses for direct oral anticoagulants 15](#_Toc115264583)

[Supplementary Table 9. Baseline characteristics stratified by the index date and oral anticoagulant type 17](#_Toc115264584)

[Supplementary Table 10. Hazard ratios and 95% confidence intervals by body mass index categories 18](#_Toc115264585)

[Supplementary Figure 1. Study design diagram 20](#_Toc115264586)

[Supplementary Figure 2. Study flow of the included patients for this study 22](#_Toc115264587)

[Supplementary Figure 3. Benefit-to-harm ratios of oral anticoagulants by body mass index categories 23](#_Toc115264588)

[Supplementary References 24](#_Toc115264589)

# Part 1 – Additional Methods

## Exposure

The daily dose was categorised as standard or lower dose than the recommended daily dose: 300 mg for dabigatran, 20 mg for rivaroxaban, 10 mg for apixaban, and 60mg for edoxaban. Since the prescription details (number of days of number of tablets per day) had missing data, it was assumed that the standard dose was prescribed when the following tablets were prescribed: 150 mg tablet for Dabigatran, 20 mg tablet for rivaroxaban, 5 mg tablet for apixaban, and 60mg tablet for edoxaban. Precise dosages for warfarin were not available because they vary according to international normalisation ratio measurement and are not routinely recorded in general practice.^1^

## Covariates

- Smoking status: the closest smoking status recorded prior to the index date in Clinical Practice Research Datalink (CPRD). We categorised as ‘ex-smokers’ patients who had non-smoker status at baseline but for whom ‘current smoking’ or ‘ex-smoking’ had been recorded at any time prior to the index date (the date of the first prescription of an oral anticoagulant after the diagnosis of atrial fibrillation during the study period).
- Presence of comorbidities: A diagnosis recorded at any time prior to the index date.
- Current medication use: ≥ 1 prescription issues for a medication in 90 days prior to the index date or on the index date.
- Beta-blockers, amiodarone, and corticosteroids were only included oral medications. Non-steroidal anti-inflammatory drugs was included oral and suppositories.

## Propensity score matching and covariates

Matching variables included age, gender, ethnicity, index of multiple deprivation, smoking, heart failure, hypertension, diabetes mellitus, myocardial infarction, peripheral artery disease, stroke, transient ischaemic attack, chronic kidney disease, gastrointestinal bleeding, cancer, dementia, depression, angiotensin converting enzyme inhibitor/angiotensin receptor blocker, beta-blockers, amiodarone, statins, proton-pump inhibitors, corticosteroids, non-steroidal anti-inflammatory drugs, and anti-platelets. We used Stata’s psmatch2-commands with 1:1 nearest neighbour matching, a maximum calliper with of 0.2 standard deviation, and without replacement.

## Missing data and multiple imputation

There are missing data in ethnicity (n=680), Index of Multiple Deprivation (IMD, n=6) and smoking status (m=111) (Supplementary Table 2 and 3). The missing-value patterns are as follows:

| Percent | Missing pattern | | |
| --- | --- | --- | --- |
|  | IMD | Smoke | Ethnicity |
| 97% | 1 | 1 | 1 |
| 2 | 1 | 1 | 0 |
| <1 | 1 | 0 | 1 |
| <1 | 0 | 1 | 1 |
| <1 | 1 | 0 | 0 |

“1” indicates that all values of the variable are not missing and “0” indicates that all values are missing.

IMD= Index of Multiple Deprivation.

97% of our data have the pattern without missing values. There are four patterns in which variables are missing. Heart failure was a significant predictor of missingness for ethnicity, proton-pomp inhibitor was a significant predictor for Index of Multiple Deprivation and chronic obstructive pulmonary disease was a significant predictor of missingness for smoking status. Therefore, we assumed to be missing at random in this cohort. As a sensitivity analysis, after unrecorded data represented by an additional missing category and propensity-score matching, missing values for ethnicity, IMD and smoking status were imputed using multiple imputation by chained equations approach, with five imputed datasets.^2^

## Benefit and harm analysis

We developed a predictive model to determine the benefit to harm ratio of oral anticoagulants (OACs) versus “Off OACs” considering that OACs might be accompanied by additional, clinically significant, serious adverse events, using a method similar to that of Phillips et al.^3^ We could simplify the risk benefit analysis by not using time varying exposure, but using the last treatment or non-treatment (“Off OACs”) as exposure prior to an outcome occurring or end of follow up. Because of the limited sample size, we combined Obesity class I and class II/III. In each propensity-matched cohort, we used major bleeding as a robust measure of harm and employed Poisson regression to determine the average number of predicted events from four models: OACs using stroke outcome and person days; OACs using major bleeding and person days; “Off OACs” using stroke outcome and person days; and “Off OACs” using major bleeding and person days. The covariates included age, sex, ethnicity and socioeconomic status (index of multiple deprivation). The analysis accounted for stroke and major bleeding experienced by a participant; recurrent events were not included in the analysis. For each of the four models, the predicted probability of stroke and major bleeding were determined for each participant. The data was randomly sampled two thirds of the data times, and each time the means of the benefit and harm were estimated. Then the benefit to harm ratio was based on the ratio of the difference in the average predicted stroke to the difference in the average predicted major bleeding in the OAC and “Off OACs” groups.

# Part 2 – Additional Results

## Supplementary Table S1. Previous observational studies reporting risk and benefit of direct oral anticoagulants in atrial fibrillation patients across body mass index categories

| Author, year | Country | Design/ Data | Population | Exposure | Comparison | Outcomes | Statistics | Size | Benefit | Risk |
| --- | --- | --- | --- | --- | --- | --- | --- | --- | --- | --- |
| Kido, 2019^4^ | USA | Single-centre, retrospective analysis | AF/AFL in morbidly obese patients* | DOACs | Warfarin | Stroke/TIA  Major bleeding | On-treatment analysis | DOACs 64; warfarin 64 | No differences | No differences |
| Lee, 2019^5^ | Korea | Korea National Health Insurance Service database | AF with BW ≤ 60kg | DOACs | Warfarin | Ischaemic stroke  Hospitalization for major bleeding | PS weighting Intention-to-treat analysis Sensitivity: switch-censored | DOACs 12810, warfarin 6692. | Lower risk with DOACs | Lower risk with DOACs |
| Peterson, 2019^6^ | USA | US healthcare claims databases | AF with morbidly obese* | Rivaroxaban | Warfarin | Ischaemic stroke/SE  Major bleeding | PS matching | 3563, each | No differences | No differences |
| Costa, 2020^7^ | USA | Electric health record data at 700 hospitals and 7000 clinics across the USA | NVAF with BMI ≥30 kg/m^2^ | Rivaroxaban | Warfarin | Stroke/SE  Major bleeding | PS matched Intention-to-treat analysis | 35,613, each | Lower risk with DOACs in patients with BMI 30.0-34.9. | Lower risk with DOACs in patients with BMI 30.0-34.9 and ≥ 40. |
| Deitelzweig, 2020^8^ | USA | ARISTOPHANES | NVAF with BMI ≥30 kg/m^2^ | Apixaban Dabigatran Rivaroxaban | Warfarin | Stroke/SE  Major bleeding | PS matched Switch-censored | 18,181 pairs for apixaban, 6646 pairs for dabigatran, and 22,053 pairs for rivaroxaban | No difference in patients with BMI ≥30 | Lower risk with apixaban and rivaroxaban in patients with BMI ≥30. No difference in patients with BMI ≥40. |
| Patti, 2020^9^ | Austria, France, Germany, Italy, Spain, Switzerland, UK, Belgium and The Netherlands. | Two prospective EU registries (PREFER in AF and PREFER in AF PROLONGATION) | AF | DOACs | VKA | Thromboembolic events  Major bleeding | PS weighting | DOAC 910, VKA 1210 | No difference in obese | No difference in obese |
| Barakat, 2021^10^ | USA | University of Pittsburgh  Medical Center hospital system | NVAF  with CHA_2_DS_2_-VASc score ≥1 | DOACs | Warfarin | Ischaemic stroke Significant bleeding | Intention-to-treat analysis | BMI <18.5 kg/m^2^, 455; 18.5 to <30, 18339; 30 to <40, 13376; ≥40, 3924 | Lower risk with DOACs across all BMI categories | Lower risk with DOACs in patients with BMI <18.5, 18.5 to <30 and ≥40 kg/m^2^  No difference in patients with BMI of 30 to <40. |

*Morbidity obese means patients with body mass index >40 kg/m^2^ or body weight >120 kg.

AF=atrial fibrillation; AFL=atrial flutter; BMI=body mass index; BW=body weight; CV=cardiovascular; DOACs=dual oral anticoagulants; ITT=intention to treat; MI=myocardial infarction; NVAF=non-valvular AF; PS=propensity score; SE=systemic embolism; VKA=Vitamin K antagonists.

## Supplementary Table S2. Baseline characteristics in whole cohort by oral anticoagulant type

|  | Warfarin | DOACs | P value |
| --- | --- | --- | --- |
| No of patients | 22818 | 6317 |  |
| Mean age (SD) | 77.5 (8.3) | 78.4 (9.2) | <0.001 |
| Women | 10218 (44.8%) | 2930 (46.4%) | 0.024 |
| Ethnicity: |  |  |  |
| White | 21948 (96.2%) | 5955 (94.3%) | 0.145 |
| Others | 420 (1.8%) | 132 (2.1%) |  |
| Missing | 450 (2.0%) | 230 (3.6%) |  |
| IMD: |  |  |  |
| 1 (Affluent) | 5353 (23.5%) | 1778 (28.1%) | <0.001 |
| 2 | 5062 (22.2%) | 1331 (21.1%) |  |
| 3 | 5195 (22.8%) | 1342 (21.2%) |  |
| 4 | 4126 (18.1%) | 1030 (16.3%) |  |
| 5 (Deprived) | 3077 (13.5%) | 835 (13.2%) |  |
| Missing | 5 (<1%) | 1 (<1%) |  |
| Smoking: |  |  |  |
| Current or ex-smoker | 14087 (61.7%) | 3814 (60.4%) | 0.089 |
| Missing | 72 (0.3%) | 39 (0.6%) |  |
| Heart Failure | 5970 (26.2%) | 1247 (19.7%) | <0.001 |
| Hypertension | 18506 (81.1%) | 5289 (83.7%) | <0.001 |
| DM | 6017 (26.4%) | 1883 (29.8%) | <0.001 |
| MI | 3416 (15.0%) | 923 (14.6%) | 0.48 |
| PAD | 2046 (9.0%) | 508 (8.0%) | 0.021 |
| Stroke | 3999 (17.5%) | 1245 (19.7%) | <0.001 |
| TIA | 2307 (10.1%) | 614 (9.7%) | 0.36 |
| COPD | 4575 (20.0%) | 1337 (21.2%) | 0.051 |
| CKD | 7923 (34.7%) | 2012 (31.9%) | <0.001 |
| GI bleeding | 3292 (14.4%) | 990 (15.7%) | 0.013 |
| Cancer | 5321 (23.3%) | 1697 (26.9%) | <0.001 |
| Dementia | 453 (2.0%) | 424 (6.7%) | <0.001 |
| Depression | 5151 (22.6%) | 1700 (26.9%) | <0.001 |
| Medications: |  |  |  |
| ACEI/ARB | 14016 (61.4%) | 3352 (53.1%) | <0.001 |
| Beta-blockers | 3438 (15.1%) | 657 (10.4%) | <0.001 |
| Amiodarone | 1172 (5.1%) | 204 (3.2%) | <0.001 |
| Statins | 12954 (56.8%) | 3474 (55.0%) | 0.012 |
| PPIs | 7494 (32.8%) | 2661 (42.1%) | <0.001 |
| Corticosteroids | 1767 (7.7%) | 597 (9.5%) | <0.001 |
| NSAIDs | 722 (3.2%) | 168 (2.7%) | 0.039 |
| Anti-platelets | 8098 (35.5%) | 3036 (48.1%) | <0.001 |

Figures are n (%) unless otherwise stated. Current medication use was prescribed within the last 90 days.

DOACs=direct oral anticoagulants; BMI=body mass index; SD=standard deviation; MI=myocardial infarction; PAD=peripheral artery disease; TIA=transient ischaemic attack; COPD=chronic obstructive pulmonary disease; CKD=chronic kidney disease; GI=gastrointestinal; ACEI/ARB=angiotensin-converting enzyme inhibitor/angiotensin receptor blocker; PPIs=proton pump inhibitors; NSAIDs=non-steroidal anti-inflammatory drugs.

## Supplementary Table S3. Baseline characteristics before matching, by BMI and oral anticoagulant type

|  | Warfarin | | | | | DOACs | | | | |
| --- | --- | --- | --- | --- | --- | --- | --- | --- | --- | --- |
| BMI categories | **Underweight**  **(< 18·5 kg/m^2^)** | **Normal weight**  **(18·5-24·9 kg/m^2^)** | **Overweight**  **(25·0-29·9 kg/m^2^)** | **Obese class I (30·0-34·9 kg/m^2^)** | **Obese class II/III (≥ 35·0 kg/m^2^)** | **Underweight**  **(< 18·5 kg/m^2^)** | **Normal weight**  **(18·5-24·9 kg/m^2^)** | **Overweight**  **(25·0-29·9 kg/m^2^)** | **Obese class I (30·0-34·9 kg/m^2^)** | **Obese class II/III (≥ 35·0 kg/m^2^)** |
| No of patients | 420 | 6585 | 8428 | 4661 | 2724 | 165 | 1842 | 2277 | 1249 | 784 |
| Mean age (SD) | 82.2 (8.0) | 80.2 (7.5) | 78.0 (7.6) | 75.5 (8.4) | 72.0 (8.7) | 83.5 (8.0) | 81.6 (8.2) | 78.5 (8.9) | 76.1 (9.0) | 72.8 (9.1) |
| Women | 302 (71.9%) | 3311 (50.3%) | 3259 (38.7%) | 1924 (41.3%) | 1422 (52.2%) | 112 (67.9%) | 936 (50.8%) | 945 (41.5%) | 531 (42.5%) | 406 (51.8%) |
| Ethnicity: |  |  |  |  |  |  |  |  |  |  |
| White | 411 (97.9%) | 6332 (96.2%) | 8116 (96.3%) | 4485 (96.2%) | 2604 (95.6%) | 161 (97.6%) | 1739 (94.4%) | 2127 (93.4%) | 1182 (94.6%) | 746 (95.2%) |
| Others | 3 (0.7%) | 123 (1.9%) | 144 (1.7%) | 94 (2.0%) | 56 (2.1%) | 2 (1.2%) | 27 (1.5%) | 67 (2.9%) | 23 (1.8%) | 13 (1.7%) |
| Missing | 6 (1.4%) | 130 (2.0%) | 168 (2.0%) | 82 (1.8%) | 64 (2.3%) | 2 (1.2%) | 76 (4.1%) | 83 (3.6%) | 44 (3.5%) | 25 (3.2%) |
| IMD: |  |  |  |  |  |  |  |  |  |  |
| 1 (Affluent) | 115 (27.4%) | 1718 (26.1%) | 2080 (24.7%) | 941 (20.2%) | 499 (18.3%) | 49 (29.7%) | 564 (30.6%) | 689 (30.3%) | 321 (25.7%) | 155 (19.8%) |
| 2 | 79 (18.8%) | 1556 (23.6%) | 1898 (22.5%) | 1019 (21.9%) | 510 (18.7%) | 32 (19.4%) | 404 (21.9%) | 506 (22.2%) | 237 (19.0%) | 152 (19.4%) |
| 3 | 100 (23.8%) | 1504 (22.8%) | 1918 (22.8%) | 1088 (23.3%) | 585 (21.5%) | 29 (17.6%) | 405 (22.0%) | 463 (20.3%) | 289 (23.1%) | 156 (19.9%) |
| 4 | 58 (13.8%) | 1091 (16.6%) | 1497 (17.8%) | 894 (19.2%) | 586 (21.5%) | 29 (17.6%) | 271 (14.7%) | 350 (15.4%) | 203 (16.3%) | 177 (22.6%) |
| 5 (Deprived) | 67 (16.0%) | 714 (10.8%) | 1034 (12.3%) | 719 (15.4%) | 543 (19.9%) | 26 (15.8%) | 198 (10.7%) | 268 (11.8%) | 199 (15.9%) | 144 (18.4%) |
| Missing | 1 (0.2%) | 2 (<1%) | 1 (<1%) | 0 (0.0%) | 1 (<1%) | 0 (0.0%) | 0 (0.0%) | 1 (<1%) | 0 (0.0%) | 0 (0.0%) |
| Smoking: |  |  |  |  |  |  |  |  |  |  |
| Current or ex-smoker | 229 (54.5%) | 3803 (57.8%) | 5247 (62.3%) | 3044 (65.3%) | 1764 (64.8%) | 99 (60.0%) | 1054 (57.2%) | 1375 (60.4%) | 798 (63.9%) | 488 (62.2%) |
| Missing | 3 (0.7%) | 20 (0.3%) | 24 (0.3%) | 18 (0.4%) | 7 (0.3%) | 0 (0.0%) | 10 (0.5%) | 16 (0.7%) | 8 (0.6%) | 5 (0.6%) |
| Heart Failure | 111 (26.4%) | 1741 (26.4%) | 2107 (25.0%) | 1210 (26.0%) | 801 (29.4%) | 37 (22.4%) | 341 (18.5%) | 432 (19.0%) | 245 (19.6%) | 192 (24.5%) |
| Hypertension | 288 (68.6%) | 4914 (74.6%) | 6822 (80.9%) | 4016 (86.2%) | 2466 (90.5%) | 121 (73.3%) | 1450 (78.7%) | 1891 (83.0%) | 1105 (88.5%) | 722 (92.1%) |
| DM | 56 (13.3%) | 1106 (16.8%) | 2062 (24.5%) | 1538 (33.0%) | 1255 (46.1%) | 21 (12.7%) | 367 (19.9%) | 610 (26.8%) | 469 (37.6%) | 416 (53.1%) |
| MI | 62 (14.8%) | 961 (14.6%) | 1318 (15.6%) | 723 (15.5%) | 352 (12.9%) | 20 (12.1%) | 263 (14.3%) | 323 (14.2%) | 202 (16.2%) | 115 (14.7%) |
| PAD | 42 (10.0%) | 624 (9.5%) | 772 (9.2%) | 409 (8.8%) | 199 (7.3%) | 20 (12.1%) | 153 (8.3%) | 173 (7.6%) | 101 (8.1%) | 61 (7.8%) |
| Stroke | 87 (20.7%) | 1275 (19.4%) | 1553 (18.4%) | 754 (16.2%) | 330 (12.1%) | 24 (14.5%) | 391 (21.2%) | 468 (20.6%) | 243 (19.5%) | 119 (15.2%) |
| TIA | 46 (11.0%) | 761 (11.6%) | 869 (10.3%) | 446 (9.6%) | 185 (6.8%) | 11 (6.7%) | 178 (9.7%) | 230 (10.1%) | 137 (11.0%) | 58 (7.4%) |
| COPD | 113 (26.9%) | 1306 (19.8%) | 1552 (18.4%) | 963 (20.7%) | 641 (23.5%) | 59 (35.8%) | 410 (22.3%) | 422 (18.5%) | 266 (21.3%) | 180 (23.0%) |
| CKD | 147 (35.0%) | 2187 (33.2%) | 2962 (35.1%) | 1682 (36.1%) | 945 (34.7%) | 52 (31.5%) | 542 (29.4%) | 728 (32.0%) | 438 (35.1%) | 252 (32.1%) |
| GI bleeding | 56 (13.3%) | 924 (14.0%) | 1243 (14.7%) | 682 (14.6%) | 387 (14.2%) | 29 (17.6%) | 302 (16.4%) | 343 (15.1%) | 203 (16.3%) | 113 (14.4%) |
| Cancer | 99 (23.6%) | 1779 (27.0%) | 1998 (23.7%) | 976 (20.9%) | 469 (17.2%) | 61 (37.0%) | 556 (30.2%) | 626 (27.5%) | 301 (24.1%) | 153 (19.5%) |
| Dementia | 20 (4.8%) | 177 (2.7%) | 160 (1.9%) | 69 (1.5%) | 27 (1.0%) | 23 (13.9%) | 159 (8.6%) | 156 (6.9%) | 61 (4.9%) | 25 (3.2%) |
| Depression | 109 (26.0%) | 1336 (20.3%) | 1792 (21.3%) | 1132 (24.3%) | 782 (28.7%) | 49 (29.7%) | 458 (24.9%) | 546 (24.0%) | 360 (28.8%) | 287 (36.6%) |
| Duration*, years: |  |  |  |  |  |  |  |  |  |  |
| Heart Failure | 4.6 (4.6) | 4.6 (5.4) | 4.8 (4.9) | 4.4 (4.7) | 4.5 (4.8) | 2.7 (4.3) | 3.9 (5.5) | 4.0 (5.0) | 4.2 (5.1) | 4.2 (5.4) |
| Hypertension | 12.1 (8.7) | 12.8 (9.5) | 12.9 (9.1) | 13.0 (9.0) | 13.5 (8.9) | 15.2 (11.9) | 14.5 (9.5) | 14.2 (9.5) | 15.2 (9.5) | 15.5 (9.3) |
| DM | 7.7 (6.4) | 9.2 (8.2) | 9.2 (7.8) | 9.0 (7.8) | 9.1 (7.3) | 9.4 (17.3) | 11.6 (8.7) | 11.3 (8.1) | 12.0 (9.2) | 11.4 (8.1) |
| MI | 8.1 (9.0) | 7.7 (7.9) | 8.0 (7.7) | 7.6 (7.4) | 7.7 (7.4) | 7.9 (11.5) | 7.7 (7.9) | 8.4 (8.6) | 8.5 (8.2) | 7.3 (7.3) |
| PAD | 7.1 (6.9) | 6.9 (6.3) | 7.5 (6.7) | 7.5 (6.5) | 6.2 (6.2) | 9.7 (8.6) | 7.7 (6.9) | 7.0 (5.9) | 7.6 (5.8) | 9.0 (7.3) |
| Stroke | 4.1 (4.6) | 3.9 (4.5) | 4.2 (4.9) | 4.0 (4.8) | 3.8 (4.7) | 1.7 (2.5) | 2.9 (4.8) | 3.2 (5.3) | 4.2 (5.9) | 3.9 (4.9) |
| TIA | 3.9 (4.2) | 4.5 (4.8) | 4.5 (5.6) | 4.7 (5.1) | 3.8 (4.1) | 2.7 (3.5) | 3.8 (4.1) | 4.2 (5.6) | 5.0 (8.3) | 4.3 (4.9) |
| COPD | 9.2 (8.3) | 8.8 (8.6) | 7.9 (7.2) | 7.7 (7.4) | 8.1 (7.5) | 9.7 (8.3) | 9.7 (8.1) | 10.0 (8.7) | 8.7 (8.8) | 9.0 (8.3) |
| CKD | 4.0 (2.7) | 4.3 (4.6) | 4.2 (4.0) | 4.1 (3.7) | 4.4 (3.9) | 6.9 (2.5) | 6.7 (3.3) | 6.8 (5.7) | 6.8 (5.4) | 7.8 (3.5) |
| GI bleeding | 8.5 (5.6) | 6.6 (5.5) | 7.4 (5.8) | 7.0 (5.2) | 6.8 (5.4) | 7.3 (6.2) | 8.5 (6.9) | 8.4 (6.5) | 8.2 (6.3) | 6.5 (5.3) |
| Cancer | 8.8 (7.3) | 7.6 (6.2) | 7.7 (7.0) | 6.5 (5.4) | 7.0 (6.6) | 14.1 (13.4) | 8.6 (8.0) | 7.9 (5.9) | 6.4 (5.2) | 8.0 (4.7) |
| Dementia | 3.0 (2.4) | 2.8 (6.9) | 1.9 (2.1) | 2.2 (2.2) | 2.0 (2.0) | 3.1 (3.1) | 2.2 (2.4) | 2.3 (2.3) | 2.1 (2.1) | 2.7 (1.5) |
| Depression | 15.8 (15.4) | 16.5 (14.8) | 16.4 (13.3) | 16.2 (13.7) | 16.1 (11.3) | 22.0 (16.0) | 16.1 (14.5) | 19.7 (15.7) | 19.3 (14.8) | 18.3 (11.8) |
| Medications: |  |  |  |  |  |  |  |  |  |  |
| ACEI/ARB | 191 (45.5%) | 3550 (53.9%) | 5210 (61.8%) | 3076 (66.0%) | 1989 (73.0%) | 59 (35.8%) | 819 (44.5%) | 1228 (53.9%) | 743 (59.5%) | 503 (64.2%) |
| Beta-blockers | 45 (10.7%) | 880 (13.4%) | 1292 (15.3%) | 765 (16.4%) | 456 (16.7%) | 12 (7.3%) | 167 (9.1%) | 245 (10.8%) | 143 (11.4%) | 90 (11.5%) |
| Amiodarone | 21 (5.0%) | 291 (4.4%) | 423 (5.0%) | 253 (5.4%) | 184 (6.8%) | 7 (4.2%) | 55 (3.0%) | 75 (3.3%) | 48 (3.8%) | 19 (2.4%) |
| Statins | 151 (36.0%) | 3288 (49.9%) | 4896 (58.1%) | 2920 (62.6%) | 1699 (62.4%) | 57 (34.5%) | 888 (48.2%) | 1291 (56.7%) | 754 (60.4%) | 484 (61.7%) |
| PPIs | 125 (29.8%) | 1987 (30.2%) | 2713 (32.2%) | 1684 (36.1%) | 985 (36.2%) | 65 (39.4%) | 781 (42.4%) | 935 (41.1%) | 521 (41.7%) | 359 (45.8%) |
| Corticosteroids | 51 (12.1%) | 518 (7.9%) | 587 (7.0%) | 387 (8.3%) | 224 (8.2%) | 24 (14.5%) | 177 (9.6%) | 182 (8.0%) | 111 (8.9%) | 103 (13.1%) |
| NSAIDs | 5 (1.2%) | 147 (2.2%) | 254 (3.0%) | 196 (4.2%) | 120 (4.4%) | 1 (0.6%) | 37 (2.0%) | 70 (3.1%) | 32 (2.6%) | 28 (3.6%) |
| Anti-platelets | 119 (28.3%) | 2193 (33.3%) | 3015 (35.8%) | 1750 (37.5%) | 1021 (37.5%) | 57 (34.5%) | 863 (46.9%) | 1089 (47.8%) | 660 (52.8%) | 367 (46.8%) |

* Duration between AF diagnosis date and index date (1^st^ prescription date of oral anticoagulant).

Figures are n (%) unless otherwise stated. Current medication use was prescribed within the last 90 days.

DOACs=direct oral anticoagulants; BMI=body mass index; SD=standard deviation; MI=myocardial infarction; PAD=peripheral artery disease; TIA=transient ischaemic attack; COPD=chronic obstructive pulmonary disease; CKD=chronic kidney disease; GI=gastrointestinal; ACEI/ARB=angiotensin-converting enzyme inhibitor/angiotensin receptor blocker; PPIs=proton pump inhibitors; NSAIDs=non-steroidal anti-inflammatory drugs.

## Supplementary Table S4. Coefficients in the propensity matching model

|  | **Underweight**  **(< 18·5 kg/m^2^)** | **Normal weight**  **(18·5-24·9 kg/m^2^)** | **Overweight**  **(25·0-29·9 kg/m^2^)** | **Obese class I**  **(30·0-34·9 kg/m^2^)** | **Obese class II/III**  **(≥ 35·0 kg/m^2^)** |
| --- | --- | --- | --- | --- | --- |
| Age | 0.030 | 0.025 | 0.003 | 0.005 | 0.012 |
| Sex | -0.130 | -0.074 | 0.090 | -0.028 | -0.179 |
| Ethnicity | -1.225 | 0.141 | -0.561 | 0.139 | 0.362 |
| IMD 1 | 0.367 | 0.162 | 0.333 | 0.206 | 0.194 |
| IMD 2 | 0.128 | -0.119 | 0.091 | -0.156 | 0.126 |
| IMD 3 | -0.160 | -0.047 | -0.009 | -0.037 | -0.005 |
| IMD 4 | 0.461 | -0.161 | -0.072 | -0.194 | 0.174 |
| Current or ex-smoker | 0.172 | -0.034 | -0.031 | -0.073 | -0.187 |
| Heart failure | -0.208 | -0.411 | -0.287 | -0.334 | -0.230 |
| Hypertension | 0.366 | 0.320 | 0.252 | 0.275 | 0.356 |
| DM | -0.227 | 0.266 | 0.165 | 0.250 | 0.349 |
| MI | -0.356 | 0.002 | -0.087 | 0.053 | 0.195 |
| PAD | 0.395 | -0.258 | -0.222 | -0.210 | -0.050 |
| Stroke | -0.462 | 0.105 | 0.094 | 0.178 | 0.223 |
| TIA | -0.530 | -0.396 | -0.123 | -0.007 | -0.048 |
| COPD | 0.287 | 0.141 | 0.014 | 0.015 | -0.155 |
| CKD | -0.180 | -0.235 | -0.186 | -0.125 | -0.202 |
| GI bleeding | 0.246 | 0.100 | -0.072 | 0.078 | -0.102 |
| Cancer | 0.680 | 0.049 | 0.166 | 0.147 | 0.078 |
| Dementia | 1.147 | 1.057 | 1.282 | 1.027 | 1.041 |
| Depression | 0.222 | 0.170 | 0.091 | 0.177 | 0.345 |
| ACEI/ARB | -0.360 | -0.342 | -0.294 | -0.275 | -0.420 |
| Beta-blockers | -0.325 | -0.452 | -0.413 | -0.441 | -0.476 |
| Amiodarone | -0.134 | -0.342 | -0.387 | -0.344 | -1.023 |
| Statins | -0.024 | -0.125 | -0.110 | -0.199 | -0.102 |
| PPIs | 0.520 | 0.462 | 0.354 | 0.131 | 0.367 |
| Corticosteroids | -0.222 | 0.069 | 0.097 | 0.018 | 0.583 |
| NSAIDs | -1.119 | -0.258 | -0.079 | -0.544 | -0.324 |

Index of multiple deprivation=5 was omitted because of collinearity.

ACEI/ARB=angiotensin-converting enzyme inhibitor/angiotensin receptor blocker; BMI=body mass index; COPD=chronic obstructive pulmonary disease; CKD=chronic kidney disease; GI=gastrointestinal; DM=diabetes mellitus; DOACs=direct oral anticoagulants; IMD= index of multiple deprivation; MI=myocardial infarction; NSAIDs=non-steroidal anti-inflammatory drugs; PAD=peripheral artery disease; PPIs=proton pump inhibitors; SD=standard deviation; TIA=transient ischaemic attack.

## Supplementary Table S5. Standardized differences before and after propensity-matching

| **BMI category** | **Underweight**  **(< 18·5 kg/m^2^)** | | | **Normal weight**  **(18·5-24·9 kg/m^2^)** | | | **Overweight**  **(25·0-29·9 kg/m^2^)** | | | **Obese class I**  **(30·0-34·9 kg/m^2^)** | | | **Obese class II/III**  **(≥ 35·0 kg/m^2^)** | | |
| --- | --- | --- | --- | --- | --- | --- | --- | --- | --- | --- | --- | --- | --- | --- | --- |
|  | **Before** | **After** | **P value (matched)** | **Before** | **After** | **P value (matched)** | **Before** | **After** | **P value (matched)** | **Before** | **After** | **P value (matched)** | **Before** | **After** | **P value (matched)** |
| Age | 17.0 | -0.9 | 0.929 | 18.8 | 0.9 | 0.777 | 6.5 | 3.8 | 0.197 | 7.5 | -2.0 | 0.614 | 9.2 | 0.5 | 0.919 |
| Sex | -8.6 | -4.0 | 0.723 | 1.0 | -1.2 | 0.717 | 5.8 | -1.1 | 0.718 | 2.5 | 1.6 | 0.685 | -0.9 | 3.8 | 0.449 |
| Ethnicity (white) | -5.1 | 0 | 1.000 | 3.1 | -1.3 | 0.672 | -8.2 | 2.3 | 0.495 | 1.3 | 3.5 | 0.401 | 2.9 | 2.8 | 0.574 |
| IMD: |  |  |  |  |  |  |  |  |  |  |  |  |  |  |  |
| 1 (Affluent) | 5.0 | 6.7 | 0.542 | 10.0 | -1.6 | 0.643 | 12.5 | -0.9 | 0.772 | 13.1 | -0.2 | 0.964 | 3.7 | -3.2 | 0.531 |
| 2 | 1.4 | -4.6 | 0.683 | -4.1 | 2.7 | 0.399 | -0.7 | -1.4 | 0.645 | -7.2 | -4.6 | 0.249 | 1.7 | 5.2 | 0.297 |
| 3 | -15.5 | 7.5 | 0.455 | -2.1 | -2.0 | 0.553 | -5.9 | 1.6 | 0.579 | -0.5 | 1.3 | 0.739 | -3.9 | 1.6 | 0.751 |
| 4 | 10.3 | -3.3 | 0.776 | -5.1 | 1.6 | 0.606 | -6.4 | 0.8 | 0.773 | -7.7 | 1.3 | 0.744 | 2.5 | -5.8 | 0.260 |
| 5 (Deprived) | -0.6 | -8.3 | 0.468 | -0.3 | -0.7 | 0.832 | -1.5 | 0 | 1.000 | 1.4 | 2.4 | 0.543 | -4.0 | 2.6 | 0.598 |
| Current or ex-smoker | 11.3 | 1.2 | 0.911 | -1.1 | 0.7 | 0.842 | -3.9 | 2.6 | 0.381 | -3.0 | -0.2 | 0.967 | -5.3 | -0.3 | 0.958 |
| Heart Failure | -9.5 | 1.4 | 0.895 | -19.1 | -0.4 | 0.899 | -14.7 | 2.9 | 0.301 | -15.2 | 0.4 | 0.920 | -11.1 | -3.7 | 0.451 |
| Hypertension | 10.6 | 2.7 | 0.805 | 9.7 | 2.4 | 0.448 | 5.5 | -2.5 | 0.380 | 6.9 | 0 | 1.000 | 5.4 | 1.4 | 0.781 |
| DM | -1.2 | 0 | 1.000 | 8.1 | 2.8 | 0.405 | 5.3 | 2.7 | 0.363 | 9.5 | -0.2 | 0.967 | 14.0 | 0.5 | 0.919 |
| MI | -7.8 | 1.8 | 0.865 | -0.9 | 2.3 | 0.475 | -4.1 | 5.9 | 0.035 | 1.8 | 2.4 | 0.546 | 5.0 | -5.2 | 0.330 |
| PAD | 6.7 | 1.9 | 0.865 | -4.1 | 1.7 | 0.586 | -5.6 | 2.4 | 0.392 | -2.5 | 3.5 | 0.365 | 1.8 | 0.5 | 0.925 |
| Stroke | -15.7 | 0 | 1.000 | 4.6 | 1.3 | 0.686 | 5.4 | -2.4 | 0.424 | 8.6 | -1.5 | 0.725 | 8.9 | -1.5 | 0.780 |
| TIA | -14.5 | -2.2 | 0.829 | -6.2 | 1.4 | 0.652 | -0.7 | 0 | 1.000 | 4.6 | 0.5 | 0.898 | 2.4 | 0 | 1.000 |
| COPD | 19.0 | 2.6 | 0.818 | 5.9 | 2.0 | 0.550 | 0.3 | 2.4 | 0.419 | 1.6 | 0.6 | 0.883 | -1.4 | -3.0 | 0.552 |
| CKD | -7.6 | 1.3 | 0.906 | -8.2 | 3.4 | 0.291 | -6.7 | 1.8 | 0.545 | -2.1 | 4.2 | 0.291 | -5.4 | -1.6 | 0.747 |
| GI bleeding | 11.6 | -1.7 | 0.886 | 6.6 | 3.0 | 0.367 | 0.9 | -0.1 | 0.967 | 4.5 | 0 | 1.000 | 0.6 | -1.1 | 0.830 |
| Cancer | 29.3 | 1.3 | 0.909 | 7.0 | 1.0 | 0.774 | 8.7 | -0.5 | 0.868 | 7.6 | -1.0 | 0.816 | 5.9 | -2.3 | 0.659 |
| Dementia | 31.8 | 10.5 | 0.406 | 25.9 | 5.4 | 0.163 | 24.4 | 6.7 | 0.056 | 19.5 | 4.6 | 0.334 | 15.4 | 4.5 | 0.450 |
| Depression | 8.2 | 0 | 1.000 | 10.9 | 1.7 | 0.619 | 6.5 | 2.5 | 0.401 | 10.3 | 0.5 | 0.894 | 16.9 | 3.5 | 0.494 |
| ACEI/ARB | -20.1 | -1.2 | 0.909 | -19.0 | 0.7 | 0.842 | -16.0 | 0.9 | 0.766 | -4.7 | 1.8 | 0.655 | -1.4 | -1.6 | 0.368 |
| Beta-blockers | -12.1 | -6.4 | 0.548 | -13.7 | -1.6 | 0.610 | -13.6 | -0.9 | 0.739 | 11.5 | 1.2 | 0.900 | 19.6 | 3.9 | 0.534 |
| Amiodarone | -3.7 | 0 | 1.000 | -7.6 | 1.7 | 0.551 | -8.6 | 2.6 | 0.300 | -9.1 | 2.2 | 0.671 | -4.3 | 1.3 | 0.284 |
| Statins | -3.1 | 1.3 | 0.908 | -3.5 | 2.1 | 0.531 | -2.8 | 4.1 | 0.170 | 31.1 | 2.0 | 0.654 | 18.9 | -4.7 | 0.755 |
| PPIs | 20.7 | 1.3 | 0.911 | 25.7 | 3.3 | 0.333 | 18.5 | 4.2 | 0.164 | 2.1 | -4.3 | 0.776 | 15.9 | -6.2 | 0.447 |
| Corticosteroids | 7.0 | -7.1 | 0.547 | 6.2 | -0.6 | 0.867 | 3.9 | -2.7 | 0.391 | -13.5 | 1.8 | 0.306 | -19.1 | -4.7 | 0.277 |
| NSAIDs | -6.2 | 0 | 1.000 | -1.6 | 2.6 | 0.388 | 0.4 | 1.8 | 0.538 | -14.4 | 0.5 | 0.510 | -15.2 | -2.9 | 0.782 |
| Anti-platelets | 13.2 | -3.9 | 0.731 | 27.9 | -2.0 | 0.553 | 24.7 | 1.3 | 0.656 | -7.5 | 1.5 | 0.631 | -20.8 | 3.7 | 0.363 |

ACEI/ARB=angiotensin-converting enzyme inhibitor/angiotensin receptor blocker; BMI = body mass index; CKD=chronic kidney disease; COPD=chronic obstructive pulmonary disease; GI=gastrointestinal; IMD = index of multiple deprivation; MI=myocardial infarction; NSAIDs=non-steroidal anti-inflammatory drugs; PAD=peripheral artery disease; PPIs=proton pump inhibitors; TIA=transient ischaemic attack.

## Supplementary Table S6. Number of events by body mass index categories

|  | **Whole** | | **Underweight**  **(< 18·5 kg/m^2^)** | | **Normal weight**  **(18·5-24·9 kg/m^2^)** | | **Overweight**  **(25·0-29·9 kg/m^2^)** | | **Obese class I**  **(30·0-34·9 kg/m^2^)** | | **Obese class II/III**  **(≥ 35·0 kg/m^2^)** | | |
| --- | --- | --- | --- | --- | --- | --- | --- | --- | --- | --- | --- | --- | --- |
|  | **Person-years** | **Events** | **Person-years** | **Events** | **Person-years** | **Events** | **Person-years** | **Events** | **Person-years** | **Events** | **Person-years** | **Events** |  |
| **Ischaemic stroke** | | | | | | | | | | | | |  |
| Warfarin | 16302 | 234 | 279 | 9 | 4265 | 70 | 6186 | 88 | 3478 | 42 | 2093 | 25 |  |
| DOACs | 9819 | 172 | 197 | 4 | 2665 | 61 | 3619 | 56 | 2077 | 38 | 1260 | 13 |  |
| Off OACs | 3613 | 134 | 96 | 5 | 1085 | 53 | 1244 | 48 | 748 | 18 | 439 | 10 |  |
| **Major bleeding** | | | | | | | | | | | | |  |
| Warfarin | 16147 | 332 | 270 | 9 | 4247 | 103 | 6109 | 121 | 3437 | 63 | 2086 | 36 |  |
| DOACs | 9812 | 219 | 195 | 12 | 2676 | 64 | 3608 | 73 | 2075 | 47 | 1259 | 23 |  |
| Off OACs | 3348 | 64 | 85 | 2 | 995 | 20 | 1148 | 21 | 704 | 13 | 414 | 8 |  |
| **All-cause mortality** | | | | | | | | | | | | |  |
| Warfarin | 16477 | 976 | 280 | 51 | 4335 | 333 | 6243 | 318 | 3511 | 151 | 2109 | 123 |  |
| DOACs | 10009 | 887 | 201 | 54 | 2730 | 317 | 3678 | 282 | 2120 | 159 | 1280 | 75 |  |
| Off OACs | 3703 | 656 | 97 | 30 | 1114 | 250 | 1274 | 221 | 767 | 97 | 451 | 58 |  |

“Off OACs” refers to patients who were prescribed oral anticoagulant but did not persist with prescriptions.

DOACs=direct oral anticoagulants; OACs=oral anticoagulants.

## Supplementary Table S7. Baseline characteristics in patients with CHA_2_DS_2_-VAS_C_ score ≥ 2 in women and ≥ 1 in men stratified by body mass index and oral anticoagulant type at the study entry after propensity score matching

|  | Warfarin | | | | | DOACs | | | | |
| --- | --- | --- | --- | --- | --- | --- | --- | --- | --- | --- |
| BMI categories | **Underweight**  **(< 18·5 kg/m^2^)** | **Normal weight**  **(18·5-24·9 kg/m^2^)** | **Overweight**  **(25·0-29·9 kg/m^2^)** | **Obese class I (30·0-34·9 kg/m^2^)** | **Obese class II/III (≥ 35·0 kg/m^2^)** | **Underweight**  **(< 18·5 kg/m^2^)** | **Normal weight**  **(18·5-24·9 kg/m^2^)** | **Overweight**  **(25·0-29·9 kg/m^2^)** | **Obese class I (30·0-34·9 kg/m^2^)** | **Obese class II/III (≥ 35·0 kg/m^2^)** |
| No of patients | 175 | 2010 | 2554 | 1417 | 930 | 175 | 2010 | 2554 | 1417 | 930 |
| Mean age (SD) | 82.8 (7.8) | 80.1 (7.8) | 76.6 (8.6) | 74.3 (8.9) | 70.2 (9.4) | 82.6 (8.7) | 80.4 (9.1) | 76.9 (9.9) | 74.4 (10.1) | 70.5 (10.4) |
| Women | 123 (70.3%) | 1023 (50.9%) | 1010 (39.5%) | 595 (42.0%) | 463 (49.8%) | 119 (68.0%) | 1018 (50.6%) | 1015 (39.7%) | 575 (40.6%) | 478 (51.4%) |
| Ethnicity (White) | 172 (98.3%) | 1974 (98.2%) | 2470 (96.7%) | 1395 (98.4%) | 921 (99.0%) | 172 (98.3%) | 1977 (98.4%) | 2481 (97.1%) | 1392 (98.2%) | 914 (98.3%) |
| IMD |  |  |  |  |  |  |  |  |  |  |
| 1 (Affluent) | 56 (32.0%) | 632 (31.4%) | 791 (31.0%) | 378 (26.7%) | 163 (17.5%) | 51 (29.1%) | 629 (31.3%) | 785 (30.7%) | 359 (25.3%) | 173 (18.6%) |
| 2 | 34 (19.4%) | 436 (21.7%) | 573 (22.4%) | 290 (20.5%) | 183 (19.7%) | 34 (19.4%) | 437 (21.7%) | 583 (22.8%) | 281 (19.8%) | 186 (20.0%) |
| 3 | 25 (14.3%) | 434 (21.6%) | 533 (20.9%) | 324 (22.9%) | 174 (18.7%) | 31 (17.7%) | 444 (22.1%) | 508 (19.9%) | 337 (23.8%) | 180 (19.4%) |
| 4 | 29 (16.6%) | 283 (14.1%) | 379 (14.8%) | 200 (14.1%) | 218 (23.4%) | 31 (17.7%) | 289 (14.4%) | 386 (15.1%) | 219 (15.5%) | 214 (23.0%) |
| 5 (Deprived) | 31 (17.7%) | 225 (11.2%) | 278 (10.9%) | 225 (15.9%) | 192 (20.6%) | 28 (16.0%) | 211 (10.5%) | 292 (11.4%) | 221 (15.6%) | 177 (19.0%) |
| Current or ex-smoker | 93 (53.1%)* | 1123 (55.9%) | 1544 (60.5%) | 897 (63.3%) | 585 (62.9%) | 102 (58.3%)* | 1145 (57.0%) | 1539 (60.3%) | 893 (63.0%) | 573 (61.6%) |
| Heart Failure | 38 (21.7%) | 349 (17.4%) | 416 (16.3%) | 230 (16.2%) | 220 (23.7%) | 37 (21.1%) | 348 (17.3%) | 449 (17.6%) | 253 (17.9%) | 201 (21.6%) |
| Hypertension | 122 (69.7%) | 1470 (73.1%) | 1952 (76.4%) | 1166 (82.3%) | 808 (86.9%) | 121 (69.1%) | 1481 (73.7%) | 1990 (77.9%) | 1186 (83.7%) | 810 (87.1%) |
| DM | 20 (11.4%) | 359 (17.9%) | 614 (24.0%) | 487 (34.4%) | 449 (48.3%) | 21 (12.0%) | 373 (18.6%) | 616 (24.1%) | 483 (34.1%) | 438 (47.1%) |
| MI | 24 (13.7%) | 238 (11.8%) | 296 (11.6%) | 200 (14.1%) | 133 (14.3%) | 21 (12.0%) | 264 (13.1%) | 332 (13.0%) | 206 (14.5%) | 116 (12.5%) |
| PAD | 13 (7.4%)* | 148 (7.4%) | 158 (6.2%) | 87 (6.1%) | 66 (7.1%) | 20 (11.4%)* | 153 (7.6%) | 175 (6.9%) | 103 (7.3%) | 61 (6.6%) |
| Stroke | 31 (17.7%)* | 387 (19.3%) | 485 (19.0%) | 254 (17.9%) | 127 (13.7%) | 24 (13.7%)* | 391 (19.5%) | 468 (18.3%) | 243 (17.1%) | 119 (12.8%) |
| TIA | 12 (6.9%) | 180 (9.0%) | 208 (8.1%) | 137 (9.7%) | 60 (6.5%) | 11 (6.3%) | 178 (8.9%) | 230 (9.0%) | 137 (9.7%) | 58 (6.2%) |
| COPD | 60 (34.3%) | 424 (21.1%) | 444 (17.4%) | 268 (18.9%) | 183 (19.7%) | 63 (36.0%) | 433 (21.5%) | 447 (17.5%) | 281 (19.8%) | 195 (21.0%) |
| CKD | 47 (26.9%) | 527 (26.2%) | 718 (28.1%) | 451 (31.8%) | 275 (29.6%) | 52 (29.7%) | 547 (27.2%) | 745 (29.2%) | 443 (31.3%) | 266 (28.6%) |
| GI bleeding | 30 (17.1%) | 296 (14.7%) | 347 (13.6%) | 215 (15.2%) | 132 (14.2%) | 29 (16.6%) | 322 (16.0%) | 371 (14.5%) | 217 (15.3%) | 127 (13.7%) |
| Cancer | 69 (39.4%) | 575 (28.6%) | 650 (25.5%) | 326 (23.0%) | 178 (19.1%) | 62 (35.4%) | 587 (29.2%) | 662 (25.9%) | 322 (22.7%) | 163 (17.5%) |
| Dementia | 19 (10.9%) | 141 (7.0%) | 129 (5.1%) | 58 (4.1%) | 21 (2.3%) | 23 (13.1%) | 160 (8.0%) | 156 (6.1%) | 62 (4.4%) | 25 (2.7%) |
| Depression | 50 (28.6%) | 487 (24.2%) | 589 (23.1%) | 375 (26.5%) | 348 (37.4%) | 51 (29.1%) | 496 (24.7%) | 614 (24.0%) | 406 (28.7%) | 342 (36.8%) |
| ACEI/ARB | 61 (34.9%) | 833 (41.4%) | 1287 (50.4%) | 794 (56.0%) | 575 (61.8%) | 59 (33.7%) | 847 (42.1%) | 1312 (51.4%) | 807 (57.0%) | 562 (60.4%) |
| Beta-blockers | 17 (9.7%)* | 159 (7.9%) | 224 (8.8%) | 172 (12.1%) | 105 (11.3%) | 12 (6.9%)* | 177 (8.8%) | 260 (10.2%) | 155 (10.9%) | 101 (10.9%) |
| Amiodarone | 8 (4.6%) | 61 (3.0%) | 76 (3.0%) | 52 (3.7%) | 16 (1.7%) | 7 (4.0%) | 59 (2.9%) | 83 (3.2%) | 55 (3.9%) | 22 (2.4%) |
| Statins | 65 (37.1%) | 899 (44.7%) | 1354 (53.0%) | 798 (56.3%) | 541 (58.2%) | 57 (32.6%) | 928 (46.2%) | 1383 (54.2%) | 810 (57.2%) | 517 (55.6%) |
| PPIs | 68 (38.9%) | 803 (40.0%) | 971 (38.0%) | 599 (42.3%) | 407 (43.8%) | 69 (39.4%) | 821 (40.8%) | 1008 (39.5%) | 575 (40.6%) | 409 (44.0%) |
| Corticosteroids | 24 (13.7%) | 177 (8.8%) | 190 (7.4%) | 114 (8.0%) | 105 (11.3%) | 26 (14.9%) | 186 (9.3%) | 195 (7.6%) | 118 (8.3%) | 113 (12.2%) |
| NSAIDs | 1 (0.6%) | 42 (2.1%) | 91 (3.6%) | 32 (2.3%) | 39 (4.2%) | 1 (0.6%) | 41 (2.0%) | 79 (3.1%) | 37 (2.6%) | 33 (3.5%) |
| Anti-platelets | 59 (33.7%) | 908 (45.2%) | 1113 (43.6%) | 672 (47.4%) | 423 (45.5%) | 58 (33.1%) | 896 (44.6%) | 1156 (45.3%) | 699 (49.3%) | 399 (42.9%) |

Figures are n (%) unless otherwise stated. Current medication use was prescribed within the last 90 days.

* Standardised differences were ≥10%.

DOACs=direct oral anticoagulants; BMI=body mass index; SD=standard deviation; MI=myocardial infarction; PAD=peripheral artery disease; TIA=transient ischaemic attack; COPD=chronic obstructive pulmonary disease; CKD=chronic kidney disease; GI=gastrointestinal; ACEI/ARB=angiotensin-converting enzyme inhibitor/angiotensin receptor blocker; PPIs=proton pump inhibitors; NSAIDs=non-steroidal anti-inflammatory drugs.

## Supplementary Table S8. Baseline characteristics on lower and standard doses for direct oral anticoagulants

| BMI category | Underweight  (< 18·5 kg/m^2^) | | Normal weight  (18·5-24·9 kg/m^2^) | | Overweight  (25·0-29·9 kg/m^2^) | | Obese class I  (30·0-34·9 kg/m^2^) | | Obese class II/III  (≥ 35·0 kg/m^2^) | |
| --- | --- | --- | --- | --- | --- | --- | --- | --- | --- | --- |
|  | **Standard dose** | **Lower dose** | **Standard dose** | **Lower dose** | **Standard dose** | **Lower dose** | **Standard dose** | **Lower dose** | **Standard dose** | **Lower dose** |
| No of patients | 64 | 101 | 1111 | 731 | 1631 | 645 | 943 | 306 | 615 | 169 |
| Mean age (SD) | 79.5 (7.4) | 86.1 (7.3) | 79.1 (8.4) | 85.5 (6.3) | 76.4 (8.7) | 83.7 (7.1) | 74.4 (8.6) | 81.4 (8.1) | 71.5 (8.7) | 77.5 (8.7) |
| Women | 32 (50.0%) | 80 (79.2%) | 492 (44.3%) | 444 (60.7%) | 625 (38.3%) | 320 (49.6%) | 363 (38.5%) | 168 (54.9%) | 299 (48.6%) | 107 (63.3%) |
| Ethnicity (White) | 62 (96.9%) | 101 (100.0%) | 1093 (98.4%) | 722 (98.8%) | 1584 (97.1%) | 625 (96.9%) | 926 (98.2%) | 300 (98.0%) | 604 (98.2%) | 167 (98.8%) |
| IMD |  |  |  |  |  |  |  |  |  |  |
| 1 (Affluent) | 16 (25.0%) | 33 (32.7%) | 351 (31.6%) | 213 (29.1%) | 519 (31.8%) | 170 (26.4%) | 255 (27.0%) | 66 (21.6%) | 130 (21.1%) | 25 (14.8%) |
| 2 | 13 (20.3%) | 19 (18.8%) | 237 (21.3%) | 167 (22.8%) | 362 (22.2%) | 144 (22.3%) | 176 (18.7%) | 61 (19.9%) | 120 (19.5%) | 32 (18.9%) |
| 3 | 10 (15.6%) | 19 (18.8%) | 249 (22.4%) | 156 (21.3%) | 324 (19.9%) | 139 (21.6%) | 214 (22.7%) | 75 (24.5%) | 122 (19.8%) | 34 (20.1%) |
| 4 | 13 (20.3%) | 16 (15.8%) | 165 (14.9%) | 106 (14.5%) | 239 (14.7%) | 111 (17.2%) | 153 (16.2%) | 50 (16.3%) | 135 (22.0%) | 42 (24.9%) |
| 5 (Deprived) | 12 (18.8%) | 14 (13.9%) | 109 (9.8%) | 89 (12.2%) | 187 (11.5%) | 81 (12.6%) | 145 (15.4%) | 54 (17.6%) | 108 (17.6%) | 36 (21.3%) |
| Current or ex-smoker | 42 (65.6%) | 57 (56.4%) | 657 (59.1%) | 397 (54.3%) | 1000 (61.3%) | 374 (58.0%) | 615 (65.2%) | 183 (59.8%) | 381 (62.0%) | 107 (63.3%) |
| Heart Failure | 9 (14.1%) | 28 (27.7%) | 184 (16.6%) | 157 (21.5%) | 281 (17.2%) | 150 (23.3%) | 155 (16.4%) | 90 (29.4%) | 143 (23.3%) | 49 (29.0%) |
| Hypertension | 46 (71.9%) | 75 (74.3%) | 834 (75.1%) | 616 (84.3%) | 1327 (81.4%) | 563 (87.3%) | 823 (87.3%) | 282 (92.2%) | 563 (91.5%) | 159 (94.1%) |
| DM | 11 (17.2%) | 10 (9.9%) | 221 (19.9%) | 146 (20.0%) | 429 (26.3%) | 181 (28.1%) | 353 (37.4%) | 116 (37.9%) | 325 (52.8%) | 91 (53.8%) |
| MI | 10 (15.6%) | 10 (9.9%) | 143 (12.9%) | 120 (16.4%) | 220 (13.5%) | 103 (16.0%) | 151 (16.0%) | 51 (16.7%) | 83 (13.5%) | 32 (18.9%) |
| PAD | 10 (15.6%) | 10 (9.9%) | 86 (7.7%) | 67 (9.2%) | 116 (7.1%) | 57 (8.8%) | 75 (8.0%) | 26 (8.5%) | 45 (7.3%) | 16 (9.5%) |
| Stroke | 6 (9.4%) | 18 (17.8%) | 222 (20.0%) | 169 (23.1%) | 329 (20.2%) | 139 (21.6%) | 179 (19.0%) | 64 (20.9%) | 84 (13.7%) | 35 (20.7%) |
| TIA | 3 (4.7%) | 8 (7.9%) | 94 (8.5%) | 84 (11.5%) | 153 (9.4%) | 77 (11.9%) | 101 (10.7%) | 36 (11.8%) | 35 (5.7%) | 23 (13.6%) |
| COPD | 28 (43.8%) | 31 (30.7%) | 234 (21.1%) | 176 (24.1%) | 292 (17.9%) | 130 (20.2%) | 193 (20.5%) | 73 (23.9%) | 133 (21.6%) | 47 (27.8%) |
| CKD | 13 (20.3%) | 39 (38.6%) | 241 (21.7%) | 301 (41.2%) | 404 (24.8%) | 324 (50.2%) | 255 (27.0%) | 183 (59.8%) | 151 (24.6%) | 101 (59.8%) |
| GI bleeding | 12 (18.8%) | 17 (16.8%) | 169 (15.2%) | 133 (18.2%) | 235 (14.4%) | 108 (16.7%) | 158 (16.8%) | 45 (14.7%) | 84 (13.7%) | 29 (17.2%) |
| Cancer | 17 (26.6%) | 44 (43.6%) | 304 (27.4%) | 252 (34.5%) | 411 (25.2%) | 215 (33.3%) | 215 (22.8%) | 86 (28.1%) | 107 (17.4%) | 46 (27.2%) |
| Dementia | 4 (6.3%) | 19 (18.8%) | 69 (6.2%) | 90 (12.3%) | 94 (5.8%) | 62 (9.6%) | 38 (4.0%) | 23 (7.5%) | 19 (3.1%) | 6 (3.6%) |
| Depression | 19 (29.7%) | 30 (29.7%) | 268 (24.1%) | 190 (26.0%) | 396 (24.3%) | 150 (23.3%) | 262 (27.8%) | 98 (32.0%) | 234 (38.0%) | 53 (31.4%) |
| ACEI/ARB | 21 (32.8%) | 38 (37.6%) | 476 (42.8%) | 343 (46.9%) | 881 (54.0%) | 347 (53.8%) | 561 (59.5%) | 182 (59.5%) | 404 (65.7%) | 99 (58.6%) |
| Beta-blockers | 4 (6.3%) | 8 (7.9%) | 97 (8.7%) | 70 (9.6%) | 168 (10.3%) | 77 (11.9%) | 102 (10.8%) | 41 (13.4%) | 72 (11.7%) | 18 (10.7%) |
| Amiodarone | 3 (4.7%) | 4 (4.0%) | 38 (3.4%) | 17 (2.3%) | 55 (3.4%) | 20 (3.1%) | 33 (3.5%) | 15 (4.9%) | 13 (2.1%) | 6 (3.6%) |
| Statins | 21 (32.8%) | 36 (35.6%) | 558 (50.2%) | 330 (45.1%) | 942 (57.8%) | 349 (54.1%) | 570 (60.4%) | 184 (60.1%) | 379 (61.6%) | 105 (62.1%) |
| PPIs | 24 (37.5%) | 41 (40.6%) | 448 (40.3%) | 333 (45.6%) | 640 (39.2%) | 295 (45.7%) | 368 (39.0%) | 153 (50.0%) | 264 (42.9%) | 95 (56.2%) |
| Corticosteroids | 11 (17.2%) | 13 (12.9%) | 106 (9.5%) | 71 (9.7%) | 121 (7.4%) | 61 (9.5%) | 79 (8.4%) | 32 (10.5%) | 79 (12.8%) | 24 (14.2%) |
| NSAIDs | 0 (0.0%) | 1 (1.0%) | 27 (2.4%) | 10 (1.4%) | 59 (3.6%) | 11 (1.7%) | 28 (3.0%) | 4 (1.3%) | 24 (3.9%) | 4 (2.4%) |
| Anti-platelets | 22 (34.4%) | 35 (34.7%) | 487 (43.8%) | 376 (51.4%) | 737 (45.2%) | 352 (54.6%) | 484 (51.3%) | 176 (57.5%) | 266 (43.3%) | 101 (59.8%) |

The daily dose was categorised as standard or lower dose than the recommended daily dose: 300 mg for dabigatran, 20 mg for rivaroxaban, 10 mg for apixaban, and 60mg for edoxaban. Figures are n (%) unless otherwise stated. Current medication use was prescribed within the last 90 days.

BMI=body mass index; SD=standard deviation; MI=myocardial infarction; PAD=peripheral artery disease; TIA=transient ischaemic attack; COPD=chronic obstructive pulmonary disease; CKD=chronic kidney disease; GI=gastrointestinal; ACEI/ARB=angiotensin-converting enzyme inhibitor/angiotensin receptor blocker; PPIs=proton pump inhibitors; NSAIDs=non-steroidal anti-inflammatory drugs.

## Supplementary Table S9. Baseline characteristics stratified by the index date and oral anticoagulant type

|  | Index date < 2015 | | Index date ≥2015 | |
| --- | --- | --- | --- | --- |
|  | **Warfarin** | **DOACs** | **Warfarin** | **DOACs** |
| No of patients | 21122 | 1117 | 1696 | 5200 |
| Mean age (SD) | 77.4 (8.3) | 78.5 (8.9) | 77.5 (8.7) | 78.3 (9.3) |
| Women | 9463 (44.8%) | 519 (46.5%) | 755 (44.5%) | 2411 (46.4%) |
| Ethnicity (white) | 20368 (96.4%) | 1075 (96.2%) | 1580 (93.2%) | 4880 (93.8%) |
| IMD: |  |  |  |  |
| 1 (Affluent) | 4898 (23.2%) | 289 (25.9%) | 455 (26.8%) | 1489 (28.6%) |
| 2 | 4703 (22.3%) | 237 (21.2%) | 359 (21.2%) | 1094 (21.0%) |
| 3 | 4844 (22.9%) | 265 (23.7%) | 351 (20.7%) | 1077 (20.7%) |
| 4 | 3814 (18.1%) | 179 (16.0%) | 312 (18.4%) | 851 (16.4%) |
| 5 (Deprived) | 2858 (13.5%) | 147 (13.2%) | 219 (12.9%) | 688 (13.2%) |
| Current or ex-smoker | 13047 (61.8%) | 664 (59.4%) | 1040 (61.3%) | 3150 (60.6%) |
| Heart Failure | 5602 (26.5%) | 222 (19.9%) | 368 (21.7%) | 1025 (19.7%) |
| Hypertension | 17086 (80.9%) | 938 (84.0%) | 1420 (83.7%) | 4351 (83.7%) |
| DM | 5503 (26.1%) | 311 (27.8%) | 514 (30.3%) | 1572 (30.2%) |
| MI | 3162 (15.0%) | 158 (14.1%) | 254 (15.0%) | 765 (14.7%) |
| PAD | 1906 (9.0%) | 82 (7.3%) | 140 (8.3%) | 426 (8.2%) |
| Stroke | 3765 (17.8%) | 284 (25.4%) | 234 (13.8%) | 961 (18.5%) |
| TIA | 2191 (10.4%) | 141 (12.6%) | 116 (6.8%) | 473 (9.1%) |
| COPD | 4251 (20.1%) | 226 (20.2%) | 324 (19.1%) | 1111 (21.4%) |
| CKD | 7356 (34.8%) | 369 (33.0%) | 567 (33.4%) | 1643 (31.6%) |
| GI bleeding | 3034 (14.4%) | 154 (13.8%) | 258 (15.2%) | 836 (16.1%) |
| Cancer | 4873 (23.1%) | 283 (25.3%) | 448 (26.4%) | 1414 (27.2%) |
| Dementia | 407 (1.9%) | 71 (6.4%) | 46 (2.7%) | 353 (6.8%) |
| Depression | 4738 (22.4%) | 269 (24.1%) | 413 (24.4%) | 1431 (27.5%) |
| Medications: |  |  |  |  |
| ACEI/ARB | 13100 (62.0%) | 629 (56.3%) | 916 (54.0%) | 2723 (52.4%) |
| Beta-blockers | 3227 (15.3%) | 157 (14.1%) | 211 (12.4%) | 500 (9.6%) |
| Amiodarone | 1112 (5.3%) | 45 (4.0%) | 60 (3.5%) | 159 (3.1%) |
| Statins | 12041 (57.0%) | 666 (59.6%) | 913 (53.8%) | 2808 (54.0%) |
| PPIs | 6860 (32.5%) | 450 (40.3%) | 634 (37.4%) | 2211 (42.5%) |
| Corticosteroids | 1611 (7.6%) | 78 (7.0%) | 156 (9.2%) | 519 (10.0%) |
| NSAIDs | 676 (3.2%) | 32 (2.9%) | 46 (2.7%) | 136 (2.6%) |
| Anti-platelets | 7327 (34.7%) | 662 (59.3%) | 771 (45.5%) | 2374 (45.7%) |

Current medication use was prescribed within the last 90 days.

BMI=body mass index; SD=standard deviation; MI=myocardial infarction; PAD=peripheral artery disease; TIA=transient ischaemic attack; COPD=chronic obstructive pulmonary disease; CKD=chronic kidney disease; GI=gastrointestinal; ACEI/ARB=angiotensin-converting enzyme inhibitor/angiotensin receptor blocker; PPIs=proton pump inhibitors; NSAIDs=non-steroidal anti-inflammatory drugs.

## Supplementary Table S10. Hazard ratios and 95% confidence intervals by body mass index categories

| BMI categories |  | Underweight  (< 18·5 kg/m^2^) | | Normal weight  (18·5-24·9 kg/m^2^) | | Overweight  (25·0-29·9 kg/m^2^) | | Obese class I  (30·0-34·9 kg/m^2^) | | Obese class II/III  (≥ 35·0 kg/m^2^) | | |  |  |
| --- | --- | --- | --- | --- | --- | --- | --- | --- | --- | --- | --- | --- | --- | --- |
|  |  | **HR (95%CI)** | **p** | **HR (95%CI)** | **p** | **HR (95%CI)** | **p** | **HR (95%CI)** | **p** | **HR (95%CI)** | **p** | | | |
| Intension-to treat analysis | | | | | | | | | | | | | |  |
| Ischaemic stroke | DOACs | 0.97 (0.23 – 4.01) | 0.962 | 0.96 (0.70 – 1.31) | 0.784 | 0.82 (0.60 – 1.12) | 0.211 | 1.06 (0.69 – 1.62) | 0.785 | 0.52 (0.27 – 1.03) | 0.061 | | | |
| Major bleeding | DOACs | 1.62 (0.61 – 4.34) | 0.336 | 1.00 (0.98 – 1.02) | 0.922 | 0.77 (0.57 – 1.03) | 0.076 | 0.99 (0.69 – 1.44) | 0.978 | 0.96 (0.57 – 1.63) | 0.888 | | | |
| All-cause mortality | DOACs | 1.04 (0.71 – 1.52) | 0.850 | 1.24 (1.07 – 1.43) | 0.004 | 1.17 (1.00 – 1.37) | 0.044 | 1.50 (1.20 – 1.87) | <0.001 | 0.89 (0.66 – 1.19) | 0.427 | | | |
| Switch-censored analysis | | | | | | | | | | | | | | |
| Ischaemic stroke | DOACs | 0.83 (0.11 – 6.49) | 0.857 | 1.00 (0.68 – 1.49) | 0.993 | 0.95 (0.62 – 1.47) | 0.822 | 1.55 (0.89 – 2.72) | 0.125 | 0.72 (0.27 – 1.93) | 0.514 | | | |
| Major bleeding | DOACs | 2.62 (0.78 – 8.78) | 0.118 | 0.78 (0.53 – 1.13) | 0.187 | 0.95 (0.68 – 1.34) | 0.781 | 1.29 (0.81 – 2.08) | 0.285 | 1.11 (0.57 – 2.18) | 0.756 | | | |
| All-cause mortality | DOACs | 1.07 (0.65 – 1.76) | 0.781 | 1.46 (1.21 – 1.76) | <0.001 | 1.42 (1.15 – 1.74) | 0.001 | 1.84 (1.37 – 2.46) | <0.001 | 0.95 (0.66 – 1.37) | 0.776 | | | |
| Using weight measured up to 3 years prior to the first OAC prescription | | | | | | | | | | | |  |  |  |
| Ischaemic stroke | DOACs | –* |  | 1.02 (0.66 – 1.58) | 0.924 | 0.95 (0.63 – 1.42) | 0.798 | 1.46 (0.84 – 2.54) | 0.183 | 1.06 (0.49 – 2.31) | 0.883 | | | |
|  | Off OACs | –* |  | 2.42 (1.52 – 3.87) | <0.001 | 2.08 (1.31 – 3.30) | 0.002 | 2.81 (1.48 – 5.33) | 0.002 | 2.79 (1.17 – 6.64) | 0.021 | | | |
| Major bleeding | DOACs | 1.12 (0.19 – 6.49) | 0.903 | 0.88 (0.59 – 1.31) | 0.527 | 0.83 (0.60 – 1.16) | 0.274 | 1.16 (0.75 – 1.79) | 0.500 | 0.76 (0.45 – 1.28) | 0.301 | | | |
|  | Off OACs | 1.56 (0.17 – 14.31) | 0.695 | 0.78 (0.41 – 1.45) | 0.426 | 0.84 (0.50 – 1.41) | 0.503 | 0.98 (0.49 – 1.96) | 0.960 | 0.77 (0.33 – 1.80) | 0.553 | | | |
| All-cause mortality | DOACs | 1.27 (0.74 – 2.17) | 0.382 | 1.44 (1.19 – 1.73) | <0.001 | 1.32 (1.09 – 1.60) | 0.005 | 1.42 (1.10 – 1.82) | 0.007 | 0.98 (0.72 – 1.34) | 0.906 | | | |
|  | Off OACs | 1.59 (0.81 – 3.11) | 0.176 | 2.96 (2.43 – 3.61) | <0.001 | 2.46 (2.01 – 3.02) | <0.001 | 2.69 (2.04 – 5.54) | <0.001 | 1.97 (1.38 – 2.81) | <0.001 | | | |
| Window period = 60 days |  |  |  |  |  |  |  |  |  |  |  | | | |
| Ischaemic stroke | DOACs | 0.60 (0.09 – 3.92) | 0.592 | 1.03 (0.72 – 1.49) | 0.857 | 0.93 (0.65 – 1.33) | 0.688 | 1.22 (0.76 – 1.97) | 0.414 | 0.72 (0.34 – 1.52) | 0.387 | | | |
|  | Off OACs | 2.19 (0.50 – 9.55) | 0.298 | 2.07 (1.44 – 2.98) | <0.001 | 2.04 (1.41 – 2.96) | <0.001 | 1.53 (0.88 – 2.65) | 0.133 | 2.19 (1.02 – 4.68) | 0.044 | | | |
| Major bleeding | DOACs | 1.85 (0.62 – 5.55) | 0.270 | 0.84 (0.60 – 1.16) | 0.286 | 0.82 (0.61 – 1.11) | 0.199 | 1.02 (0.69 – 1.52) | 0.918 | 1.01 (0.58 – 1.75) | 0.974 | | | |
|  | Off OACs | 1.31 (0.29 – 5.86) | 0.725 | 0.71 (0.46 – 1.08) | 0.107 | 0.75 (0.50 – 1.12) | 0.156 | 0.95 (0.57 – 1.61) | 0.858 | 0.95 (0.45 – 2.00) | 0.900 | | | |
| All-cause mortality | DOACs | 1.40 (0.92 – 2.15) | 0.118 | 1.48 (1.25 – 1.75) | <0.001 | 1.47 (1.23 – 1.75) | <0.001 | 2.14 (1.67 – 2.74) | <0.001 | 1.01 (0.74 – 1.39) | 0.939 | | | |
|  | Off OACs | 2.13 (1.32 – 3.42) | 0.002 | 2.79 (2.37 – 3.29) | <0.001 | 3.44 (2.91 – 4.08) | <0.001 | 3.32 (2.59 – 4.27) | <0.001 | 2.52 (1.87 – 3.39) | <0.001 | | | |
| CHA_2_DS_2_-VAS_C_ score ≥ 2 in women and ≥ 1 in men | | | | | | | | | | | |  |  |  |
| Ischaemic stroke | DOACs | 1.21 (0.18 – 8.26) | 0.849 | 1.21 (0.85 – 1.74) | 0.291 | 0.83 (0.59 – 1.18) | 0.308 | 1.10 (0.70 – 1.74) | 0.683 | 1.01 (0.51 – 2.02) | 0.975 | | | |
|  | Off OACs | 2.00 (0.27 – 14.7) | 0.497 | 2.89 (1.97 – 4.25) | <0.001 | 2.32 (1.56 – 3.44) | <0.001 | 2.77 (1.63 – 4.69) | <0.001 | 2.63 (1.13 – 6.12) | 0.025 | | | |
| Major bleeding | DOACs | 1.80 (0.69 – 4.73) | 0.231 | 0.96 (0.69 – 1.34) | 0.824 | 1.05 (0.78 – 1.40) | 0.767 | 1.29 (0.88 – 1.89) | 0.190 | 0.85 (0.52 – 1.39) | 0.519 | | | |
|  | Off OACs | 0.57 (0.10 – 3.13) | 0.519 | 0.83 (0.51 – 1.36) | 0.461 | 1.20 (0.79 – 1.83) | 0.400 | 1.10 (0.62 – 1.93) | 0.753 | 0.42 (0.16 – 1.08) | 0.072 | | | |
| All-cause mortality | DOACs | 1.28 (0.85 – 1.93) | 0.230 | 1.48 (1.26 – 1.73) | <0.001 | 1.34 (1.14 – 1.58) | <0.001 | 1.67 (1.33 – 2.08) | <0.001 | 0.92 (0.69 – 1.23) | 0.572 | | | |
|  | Off OACs | 1.73 (1.04 – 2.88) | 0.036 | 2.80 (2.37 – 3.31) | <0.001 | 2.85 (2.39 – 3.41) | <0.001 | 2.94 (2.30 – 3.76) | <0.001 | 2.08 (1.52 – 2.85) | <0.001 | | | |
| Dose analysis | | | | | | | | | | | |  |  |  |
| Ischaemic stroke | Standard dose | 1.18 (0.10 – 13.4) | 0.894 | 0.96 (0.61 – 1.52) | 0.860 | 0.84 (0.56 – 1.28) | 0.426 | 1.44 (0.87 – 2.39) | 0.157 | 0.92 (0.42 – 1.99) | 0.828 | | | |
|  | Lower dose | 0.38 (0.05 – 2.67) | 0.329 | 1.23 (0.80 – 1.89) | 0.344 | 1.06 (0.66 – 1.70) | 0.817 | 1.01 (0.52 – 1.98) | 0.969 | 0.28 (0.07 – 1.20) | 0.087 | | | |
|  | Off OACs | 1.53 (0.34 – 6.88) | 0.578 | 2.53 (1.73 – 3.69) | <0.001 | 2.42 (1.63 – 3.59) | <0.001 | 2.03 (1.14 – 3.59) | 0.015 | 1.89 (0.81 – 4.41) | 0.139 | | | |
| Major bleeding | Standard dose | 0.74 (0.11 – 4.98) | 0.758 | 0.84 (0.57 – 1.25) | 0.390 | 0.81 (0.57 – 1.15) | 0.245 | 1.13 (0.72 – 1.75) | 0.602 | 0.85 (0.45 – 1.60) | 0.613 | | | |
|  | Lower dose | 1.92 (0.59 – 6.23) | 0.280 | 0.85 (0.55 – 1.30) | 0.448 | 0.93 (0.62 – 1.41) | 0.745 | 0.98 (0.51 – 1.61) | 0.728 | 1.33 (0.63 – 2.82) | 0.450 | | | |
|  | Off OACs | 0.71 (0.13 – 3.94) | 0.697 | 0.71 (0.43 – 1.16) | 0.166 | 0.77 (0.48 – 1.23) | 0.270 | 0.87 (0.47 – 1.60) | 0.660 | 1.20 (0.52 – 2.80) | 0.665 | | | |
| All-cause mortality | Standard dose | 1.16 (0.62 – 2.17) | 0.648 | 1.31 (1.06 – 1.62) | 0.011 | 1.29 (1.04 – 1.59) | 0.019 | 1.57 (1.18 – 2.10) | 0.002 | 0.78 (0.53 – 1.14) | 0.195 | | | |
|  | Lower dose | 1.48 (0.93 – 2.35) | 0.096 | 1.56 (1.30 – 1.88) | <0.001 | 1.54 (1.26 – 1.88) | <0.001 | 2.30 (1.74 – 3.04) | <0.001 | 1.35 (0.92 – 1.97) | 0.122 | | | |
|  | Off OACs | 1.83 (1.10 – 3.05) | 0.020 | 2.48 (2.09 – 2.94) | <0.001 | 3.17 (2.66 – 3.79) | <0.001 | 2.79 (2.14 – 3.63) | <0.001 | 2.19 (1.58 – 3.03) | <0.001 | | | |
| Index date ≥2015 | | | | | | | | | | | |  |  |  |
| Ischaemic stroke | DOACs | –* |  | 1.89 (0.66 – 5.42) | 0.237 | 0.71 (0.23 – 2.19) | 0.548 | –* |  | –* |  | | | |
|  | Off OACs | –* |  | 5.96 (2.22 – 15.98) | <0.001 | 6.02 (1.78 – 20.37) | 0.004 | –* |  | –* |  | | | |
| Major bleeding | DOACs | –* |  | 0.80 (0.32 – 2.02) | 0.637 | 1.13 (0.59 – 2.15) | 0.711 | 1.35 (0.57 – 3.20) | 0.496 | 0.59 (0.20 – 1.73) | 0.338 | | | |
|  | Off OACs | –* |  | 0.84 (0.18 – 3.86) | 0.820 | 0.54 (0.12 – 2.44) | 0.422 | 1.91 (0.49 – 7.46) | 0.349 | 1.41 (0.30 – 6.62) | 0.666 | | | |
| All-cause mortality | DOACs | –* |  | 1.29 (0.85 – 1.96) | 0.228 | 0.97 (0.65 – 1.43) | 0.865 | 1.45 (0.86 – 2.45) | 0.168 | 1.45 (0.68 – 3.07) | 0.387 | | | |
|  | Off OAC | –* |  | 2.49 (1.48 – 4.21) | 0.001 | 2.20 (1.35 – 3.61) | 0.002 | 2.67 (1.26 – 5.67) | 0.011 | 2.39 (0.86 – 6.63) | 0.094 | | | |
| Multiple imputation for missing data (ethnicity, index of multiple deprivation, and smoking) after propensity-score matching | | | | | | | | | | | | | | |
| Ischaemic stroke | DOACs | 0.74 (0.14 – 3.99) | 0.728 | 1.01 (0.71 – 1.42) | 0.970 | 0.90 (0.63 – 1.28) | 0.549 | 1.42 (0.89 – 2.26) | 0.145 | 0.79 (0.40 – 1.57) | 0.501 | | | |
|  | Off OACs | 1.94 (0.46 – 8.10) | 0.365 | 2.33 (1.60 – 3.39) | <0.001 | 2.71 (1.85 – 3.95) | <0.001 | 2.39 (1.36 – 4.22) | 0.003 | 1.83 (0.78 – 4.28) | 0.164 | | | |
| Major bleeding | DOACs | 1.34 (0.47 – 3.80) | 0.588 | 0.97 (0.70 – 1.35) | 0.862 | 0.83 (0.61 – 1.11) | 0.212 | 1.21 (0.82 – 1.79) | 0.345 | 0.96 (0.57 – 1.62) | 0.869 | | | |
|  | Off OACs | 0.55 (0.09 – 3.27) | 0.507 | 1.00 (0.63 – 1.61) | 0.991 | 0.75 (0.47 – 1.20) | 0.235 | 1.08 (0.60 – 1.93) | 0.805 | 1.05 (0.45 – 2.44) | 0.912 | | | |
| All-cause mortality | DOACs | 1.40 (0.79 – 2.49) | 0.247 | 1.33 (1.11 – 1.59) | 0.002 | 1.45 (1.20 – 1.75) | <0.001 | 1.42 (1.10 – 1.83) | 0.007 | 0.96 (0.70 – 1.31) | 0.791 | | | |
|  | Off OAC | 1.28 (0.60 – 2.72) | 0.526 | 2.70 (2.24 – 3.26) | <0.001 | 2.94 (2.42 – 3.58) | <0.001 | 2.40 (1.82 – 3.17) | <0.001 | 2.37 (1.67 – 3.37) | <0.001 | | | |
| Patients without interventional/surgical procedures (ablation, left atrial appendage closure or surgical left appendage removal) for atrial fibrillation at baseline | | | | | | | | | | | | | | |
| Ischaemic stroke | DOACs | 0.49 (0.09 – 2.80) | 0.422 | 1.09 (0.77 – 1.55) | 0.629 | 0.91 (0.64 – 1.30) | 0.616 | 1.28 (0.80 – 2.06) | 0.301 | 0.73 (0.35 – 1.50) | 0.385 | | | |
|  | Off OACs | 1.52 (0.34 – 6.87) | 0.585 | 2.51 (1.72 – 3.66) | <0.001 | 2.41 (1.63 – 3.58) | <0.001 | 2.10 (1.18 – 3.72) | 0.011 | 2.03 (0.86 – 4.77) | 0.104 | | | |
| Major bleeding | DOACs | 1.48 (0.55 – 3.96) | 0.432 | 0.84 (0.61 – 1.15) | 0.273 | 0.86 (0.64 – 1.15) | 0.309 | 1.03 (0.70 – 1.52) | 0.863 | 0.99 (0.57 – 1.71) | 0.972 | | | |
|  | Off OACs | 0.70 (0.13 – 3.78) | 0.676 | 0.72 (0.44 – 1.18) | 0.190 | 0.77 (0.48 – 1.23) | 0.277 | 0.88 (0.48 – 1.61) | 0.675 | 1.06 (0.43 – 2.62) | 0.892 | | | |
| All-cause mortality | DOACs | 1.37 (0.91 – 2.05) | 0.134 | 1.43 (1.23 – 1.68) | <0.001 | 1.40 (1.19 – 1.66) | <0.001 | 1.89 (1.50 – 2.38) | <0.001 | 1.00 (0.74 – 1.34) | 0.988 | | | |
|  | Off OAC | 1.80 (1.09 – 2.99) | 0.023 | 2.45 (2.06 – 2.90) | <0.001 | 3.17 (2.65 – 3.79) | <0.001 | 2.76 (2.12 – 3.58) | <0.001 | 2.21 (1.60 – 3.07) | <0.001 | | | |

The reference is warfarin. “Off OACs” refers to patients who were prescribed oral anticoagulants but did not persist with prescriptions. These were adjusted for age, gender, index of multiple deprivation, smoking, heart failure, hypertension, diabetes, myocardial infarction, peripheral artery disease, stroke, transient ischaemic attack, chronic kidney disease, gastrointestinal bleeding, cancer, dementia, depression, angiotensin converting enzyme inhibitor/angiotensin receptor blocker, beta-blockers, amiodarone, statins, proton-pump inhibitors, corticosteroids, non-steroidal anti-inflammatory drugs, and anti-platelets. For ischaemic stroke and major bleeding informative censoring of survival time was taken into account for those who died as a competing risk using Fine and Gray’s proportional sub-hazards model, to estimate cause-specific hazard ratios and 95% confidence intervals.

* Insufficient data for analysis.

BMI=body mass index; CI=confidence interval; DOACs=direct oral anticoagulants; HR=hazard ratio; OACs=oral anticoagulants.

## Supplementary Figure S1. Study design diagram


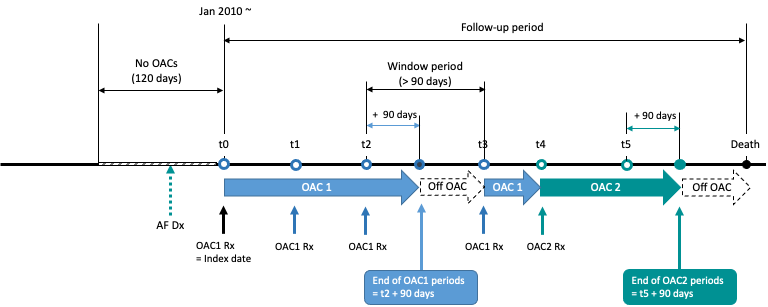


“Off OACs” refers to patients who were prescribed OACs but did not persist with prescriptions.

AF=atrial fibrillation; Dx=diagnosis; OACs=oral anticoagulants; Rx=prescription.

In scenario 1 the patient is prescribed OAC1 during the entire study period until the outcome. In this case the outcome is apportioned to time on treatment with OAC1.

In scenario 2 the patient stops receiving prescriptions of OAC1. After a 90 day period since that last OAC1 prescription date they are classified as “off OAC”. The outcome that occurs subsequently is counted towards the “off OAC” time period.


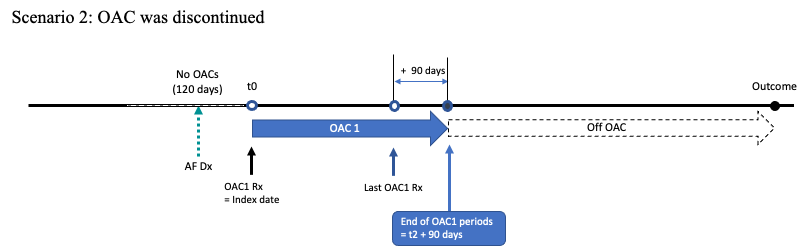


In scenario 3 the patient originally receives prescription for OAC1 but then receives prescriptions for OAC2. The subsequent outcome is apportioned to time on treatment OAC2, not OAC1.

In scenario 4 the patient the patient originally receives prescription for OAC1 but then receives prescriptions for OAC2 within 90 days of their last OAC1 prescription (i.e. “overlap”). The time from the first OAC2 prescription is apportioned to time on treatment with OAC2. If an outcome had occurred before the first OAC2 prescription, such as a bleed or stroke, it would be apportioned to time on treatment with OAC1.


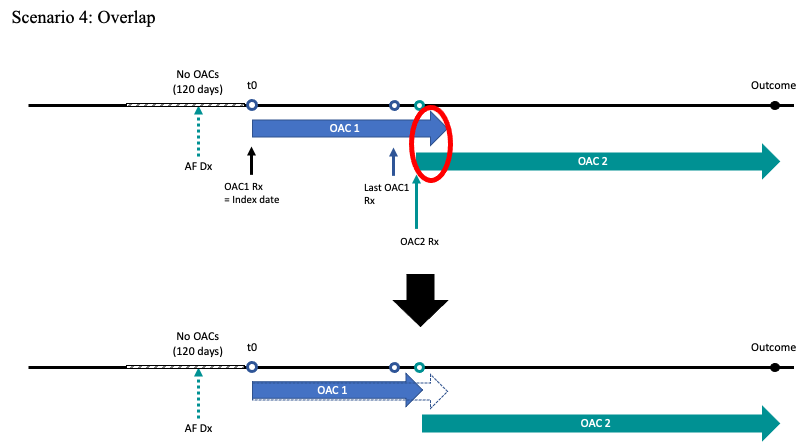


## Supplementary Figure S2. Study flow of the included patients for this study


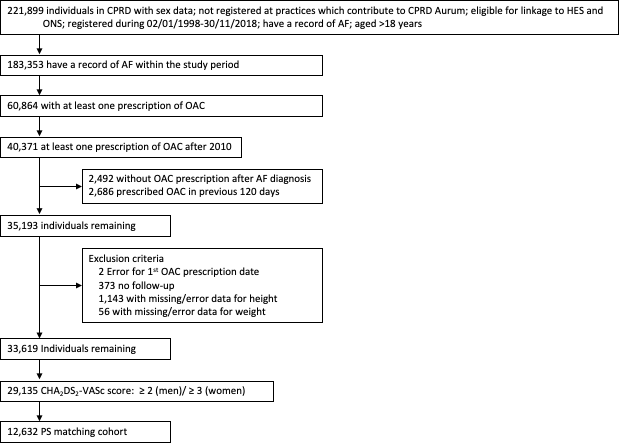


CPRD=Clinical Practice Research Datalink; HES= Hospital Episode Statistics; ONS=The Office for National Statistic; AF=atrial fibrillation; OACs=oral anticoagulants.

## Supplementary Figure S3. Benefit-to-harm ratios of oral anticoagulants by body mass index categories

Tehe ratios >1 indicate positive net benefit.

BMI=body mass index; CI=confidence interval; OAC=oral anticoagulants.

# Supplementary References

1. Vinogradova Y, Coupland C, Hill T, Hippisley-Cox J. Risks and benefits of direct oral anticoagulants versus warfarin in a real world setting: cohort study in primary care. *BMJ* 2018; **362**: k2505.

2. Perkins NJ, Cole SR, Harel O, et al. Principled Approaches to Missing Data in Epidemiologic Studies. *Am J Epidemiol* 2018; **187**(3): 568-75.

3. Phillips RA, Xu J, Peterson LE, Arnold RM, Diamond JA, Schussheim AE. Impact of Cardiovascular Risk on the Relative Benefit and Harm of Intensive Treatment of Hypertension. *J Am Coll Cardiol* 2018; **71**(15): 1601-10.

4. Kido K, Ngorsuraches S. Comparing the Efficacy and Safety of Direct Oral Anticoagulants With Warfarin in the Morbidly Obese Population With Atrial Fibrillation. *Ann Pharmacother* 2019; **53**(2): 165-70.

5. Lee SR, Choi EK, Park CS, et al. Direct Oral Anticoagulants in Patients With Nonvalvular Atrial Fibrillation and Low Body Weight. *J Am Coll Cardiol* 2019; **73**(8): 919-31.

6. Peterson ED, Ashton V, Chen YW, Wu B, Spyropoulos AC. Comparative effectiveness, safety, and costs of rivaroxaban and warfarin among morbidly obese patients with atrial fibrillation. *Am Heart J* 2019; **212**: 113-9.

7. Costa OS, Beyer-Westendorf J, Ashton V, et al. Effectiveness and safety of rivaroxaban versus warfarin in obese nonvalvular atrial fibrillation patients: analysis of electronic health record data. *Curr Med Res Opin* 2020; **36**(7): 1081-8.

8. Deitelzweig S, Keshishian A, Kang A, et al. Effectiveness and Safety of Oral Anticoagulants among NVAF Patients with Obesity: Insights from the ARISTOPHANES Study. *J Clin Med* 2020; **9**(6).

9. Patti G, Pecen L, Manu MC, et al. Thromboembolic and bleeding risk in obese patients with atrial fibrillation according to different anticoagulation strategies. *Int J Cardiol* 2020; **318**: 67-73.

10. Barakat AF, Jain S, Masri A, et al. Outcomes of Direct Oral Anticoagulants in Atrial Fibrillation Patients Across Different Body Mass Index Categories. *JACC Clin Electrophysiol* 2021.

11. Benchimol EI, Smeeth L, Guttmann A, et al. The REporting of studies Conducted using Observational Routinely-collected health Data (RECORD) Statement. *PLOS Medicine* 2015; **12**(10): e1001885.
